# Contrasting Growth and Physiological Response Profiles of Three Native Alpine *Elymus* Forage Grasses Under Combined Cadmium and PEG-6000-Induced Osmotic Stress

**DOI:** 10.3390/plants15162434

**Published:** 2026-08-10

**Authors:** Yanshuang Liu, Huichun Wang, Xuzhe Cui, Nian Liu, Qunying Chen, Lina Liu, Mingyuan Zhang

**Affiliations:** 1 College of Life Sciences, Qinghai Normal University, Xining 810008, China; 15004215046@163.com (Y.L.);; 2 Key Laboratory of Medicinal Animal and Plant Resources on the Qinghai-Tibet Plateau, Xining 810099, China; 3 Academy of Plateau Science and Sustainability, Qinghai Normal University, Xining 810016, China; 4 Qilian Mountain Southern Slope Forest Ecosystem Research Station, Huzhu 810500, China

**Keywords:** cadmium stress, PEG-6000-induced osmotic stress, combined abiotic stress, *Elymus*, antioxidant enzymes, photosynthetic pigments, principal component analysis, multi-trait response evaluation

## Abstract

The co-occurrence of soil drought and cadmium (Cd) pollution represents an important combined-stress scenario in degraded alpine grasslands, yet comparative responses of native *Elymus* forage grasses remain poorly resolved. Seedlings of *E. sibiricus* cv. Chuancao, *E. breviaristatus*, and *E. nutans* cv. Gannan were exposed to a full-factorial hydroponic matrix of four nominal Cd concentrations (0, 5, 10, and 20 mg·L^−1^) and four PEG-6000 concentrations of 0, 5, 7.5, and 10% (*w*/*v*). Growth, biomass allocation, tissue water status, malondialdehyde (MDA), soluble sugars, antioxidant enzyme activities, and photosynthetic pigments were measured, and stress tolerance coefficient, membership function analysis, and principal component analysis were used for integrated evaluation. Cd × PEG interactions affected growth, biomass, MDA, soluble sugars, peroxidase, catalase (CAT), and total chlorophyll, demonstrating trait- and species-dependent non-additive responses. Under the most severe combined treatment, *E. breviaristatus* maintained comparatively better shoot growth, lower absolute MDA, and relatively stable CAT and pigment traits; *E. nutans* maintained CAT and chlorophyll concentration but showed severe membrane lipid peroxidation, whereas *E. sibiricus* was more sensitive in shoot growth and pigment maintenance. Multi-method evaluation ranked *E. breviaristatus* first for relative multi-trait performance. Because Cd availability, tissue Cd concentrations, and root-to-shoot translocation were not measured, this ranking reflects phenotype-based performance under common external treatments rather than intrinsic Cd tolerance. *E. breviaristatus* should therefore be regarded as a candidate for subsequent Cd-accumulation, soil-pot, and field validation.

## 1. Introduction

Drought and heavy-metal pollution are major abiotic constraints on alpine plant development, grassland productivity, and ecological restoration. Drought restricts water uptake, cell elongation, photosynthetic carbon fixation, and dry-matter accumulation [1]. Cadmium (Cd) is a non-essential toxic metal that can be absorbed by roots and transported to shoots, where it promotes reactive oxygen species (ROS) accumulation, membrane lipid peroxidation, chlorophyll degradation, and nutrient imbalance [2]. Cd and other trace elements have been detected in soil–herbage systems in subalpine grasslands in parts of the northern Tibet Plateau [3]. Responses to stress combinations cannot be inferred directly from responses to individual stresses because interactions may be synergistic, antagonistic, or additive [4].

Cd toxicity and PEG-6000-induced osmotic stress affect overlapping physiological processes, including redox homeostasis, osmotic adjustment, and photosynthetic function [2,5,6]. Polyethylene glycol 6000 (PEG-6000) is widely used to impose controlled osmotic stress in laboratory systems and can rapidly induce water-deficit-related signals at the seedling stage [5]. Studies on *Passiflora edulis* under PEG-6000-induced osmotic stress and on adzuki bean under combined Cd and water-deficit stress have shown that these treatments can reduce growth and biomass while increasing MDA accumulation and altering osmolytes and antioxidant enzyme activities [5,6]. The activation of individual defense components does not necessarily prevent growth inhibition or oxidative injury under combined stress [4,6]. Consequently, no single trait can adequately represent combined-stress performance, and integrated evaluation using stress tolerance coefficients, membership functions, and principal component analysis (PCA) should be interpreted together with individual physiological traits.

Alpine grasslands on the Qinghai–Tibet Plateau provide important ecosystem and livestock-production functions [7]. Drought exerts widespread but spatially heterogeneous effects on alpine grasslands across the Plateau [7]. Together with the detection of Cd and other trace elements in regional soil–herbage systems [3], this evidence suggests that some alpine and subalpine grasslands may experience overlapping water-deficit and trace-element exposure pressures [3,7]. *Elymus nutans* is a dominant perennial forage grass in alpine meadows with high forage and ecological-restoration value, and its phenotypic and genetic differentiation under regional environmental regulation provides a basis for germplasm evaluation and stress-resistance breeding [8]. *Elymus sibiricus* is an important forage grass used in artificial grasslands on the Qinghai–Tibet Plateau [9]. *Elymus breviaristatus* is endemic to the southeastern Qinghai–Tibet Plateau and has distinctive regional conservation value as a germplasm resource [10].

Based on these considerations, the present study selected three alpine *Elymus* grasses, *E. sibiricus* cv. Chuancao, *E. breviaristatus* and *E. nutans* cv. Gannan, as experimental materials. To fill these research gaps, a full-factorial hydroponic trial was designed, and three hypotheses were proposed: (1) Cd and PEG-6000-induced osmotic stress interact significantly, and high Cd combined with severe PEG-6000-induced osmotic stress synergistically aggravates membrane lipid peroxidation; (2) the three grasses exhibit species-specific responses to combined stress, and materials showing more favorable relative performance maintain better shoot growth and biomass accumulation, lower MDA accumulation, and more stable antioxidant enzyme activity and photosynthetic pigment systems; and (3) an integrated multi-trait response score incorporating principal component analysis (PCA) can effectively compare relative multi-trait performance to combined stress, but the integrated evaluation must be interpreted together with individual growth and physiological traits.

Most existing studies on combined Cd and water-deficit stress, including soil-drought and PEG-based osmotic treatments, have mainly focused on field crops and model herbaceous plants, whereas comparative physiological studies of plateau perennial *Elymus* grasses remain scarce. Previous studies have shown that combined Cd and water-deficit treatments can cause greater growth inhibition, oxidative damage, and photosynthetic impairment than either stress alone, although the response patterns differ markedly among species. The innovation of this study lies in the use of a unified Cd × PEG factorial design to simultaneously compare growth maintenance, biomass allocation, water homeostasis, oxidative defense and photosynthetic pigment responses across three native forage grasses, thereby characterizing contrasting response profiles of alpine *Elymus* grasses. Accordingly, the present study was designed as a controlled, phenotype-based comparison of the relative seedling responses of three alpine *Elymus* grasses under a common Cd × PEG-6000 treatment matrix. We aimed to characterize interspecific differences in growth maintenance, biomass allocation, water status, membrane lipid peroxidation, osmotic adjustment, antioxidant enzyme activities, and photosynthetic pigment traits, and to identify candidate material for subsequent mechanistic validation. Because Cd availability in the nutrient solution, root and shoot Cd concentrations and total contents, and root-to-shoot translocation were not quantified, the present study was not designed to distinguish Cd exclusion from internal tissue tolerance or to establish field restoration suitability. Its conclusions are therefore restricted to comparative multi-trait performance under the specific hydroponic seedling conditions used here. The Cd × PEG-6000 matrix was designed as a graded and reproducible hydroponic screening system for standardized interspecific comparison.

## 2. Results

### 2.1. Integrated Effects of Grass Species, Cd Stress and PEG-6000-Induced Osmotic Stress

Three-way ANOVA showed that grass material, Cd concentration and PEG-6000 concentration jointly affected growth morphology, biomass allocation, water status, oxidative damage, osmotic adjustment, antioxidant enzyme activities and photosynthetic pigment composition in the three grasses. Overall, PEG-6000-induced osmotic stress exerted a stronger inhibitory effect on shoot growth. Taking plant height as an example, the main effect of PEG-6000 concentration was the strongest (*F*_3_,_96_ = 132.61, *p* < 0.001, $\eta_{p}^{2}$ = 0.806), markedly greater than the main effect of Cd (*F*_3_,_96_ = 3.28, *P* = 0.024, $\eta_{p}^{2}$ = 0.093), indicating that the water-deficit signal was the primary factor limiting seedling elongation. As treatment intensity increased, stress effects expanded from morphological traits such as plant height and root length to shoot and root dry-matter accumulation, tissue water content, MDA accumulation, changes in soluble sugars, regulation of peroxidase (POD) and CAT activities, and total chlorophyll content.

Regarding combined-treatment effects, the joint action of Cd and PEG-6000 significantly affected key traits including plant height, maximum root length, shoot dry weight, root dry weight, MDA, soluble sugars, POD, CAT and total chlorophyll. This indicates that trait responses under combined stress cannot be simply inferred from responses to single Cd stress or single PEG-6000 stress. Species specificity was particularly pronounced for MDA and soluble sugars. The three-way Grass × Cd × PEG interaction was highly significant for MDA (*F*_18_,_96_ = 42.78, *p* < 0.001, $\eta_{p}^{2}$ = 0.889) and was also very strong for soluble sugars (*F*_18_,_96_ = 170.99, *p* < 0.001, $\eta_{p}^{2}$ = 0.970), demonstrating clear differences among the three grasses in membrane lipid peroxidation and osmotic adjustment.

**Figure 1:**
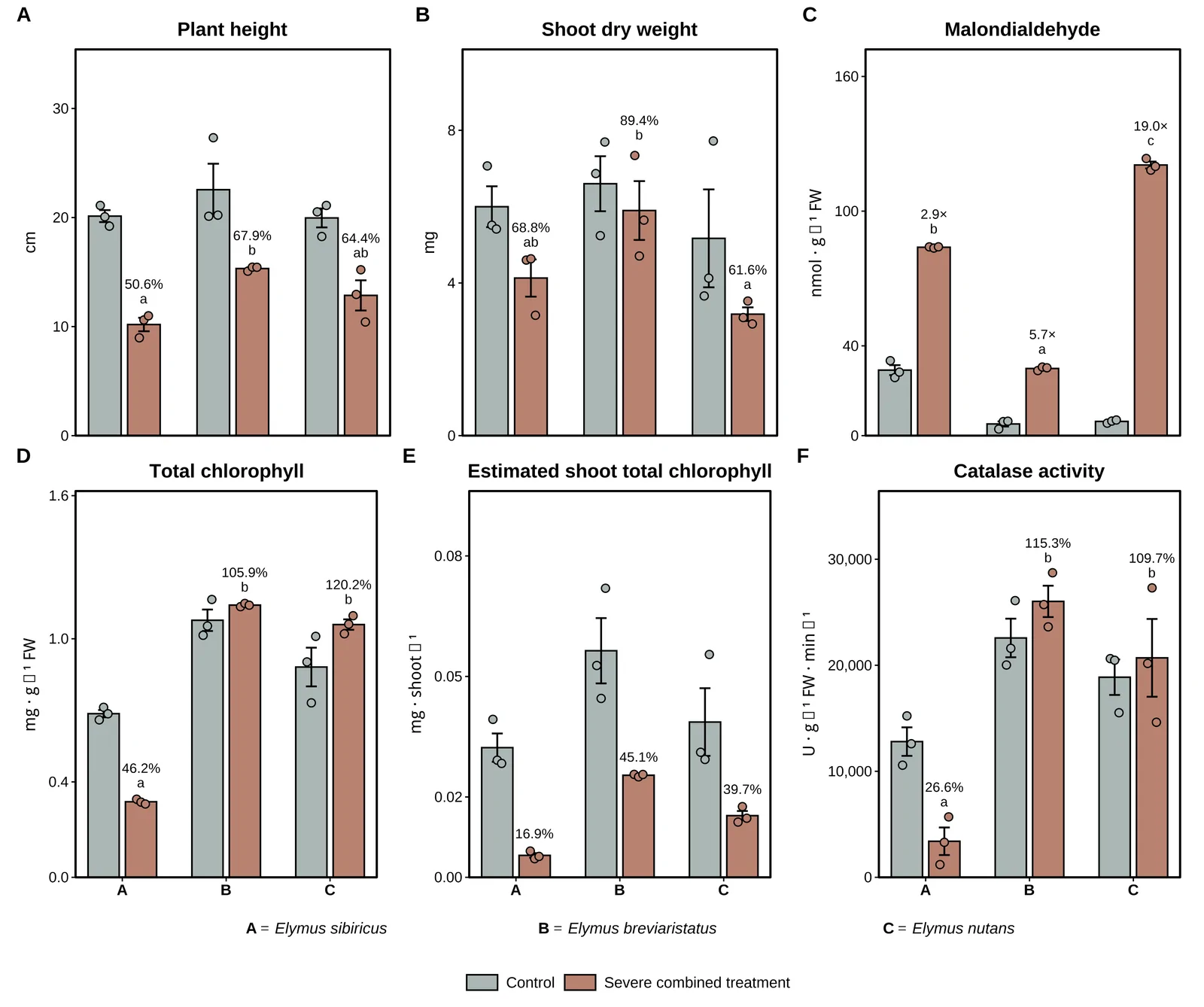
Comparison of key growth and physiological responses of three *Elymus* grasses under the control and severe combined-stress treatments. The severe combined treatment consisted of 20 mg·L^−1^ Cd and 10% (*w*/*v*) PEG-6000. (**A**) Plant height; (**B**) shoot dry weight; (**C**) malondialdehyde content; (**D**) total chlorophyll concentration; (**E**) estimated shoot total chlorophyll amount; and (**F**) catalase activity. Values are means ± SE (n = 3). Percentages or fold-change values above the severe-treatment symbols indicate responses relative to the corresponding species-specific control. Different lowercase letters indicate significant differences among the three grass species under the severe combined treatment based on estimated marginal means with Benjamini–Hochberg correction at *p* < 0.05. Estimated shoot total chlorophyll amount was calculated from replicate-level data as total chlorophyll concentration × shoot fresh weight/1000 and was used for descriptive interpretation only.

**Figure 2:**
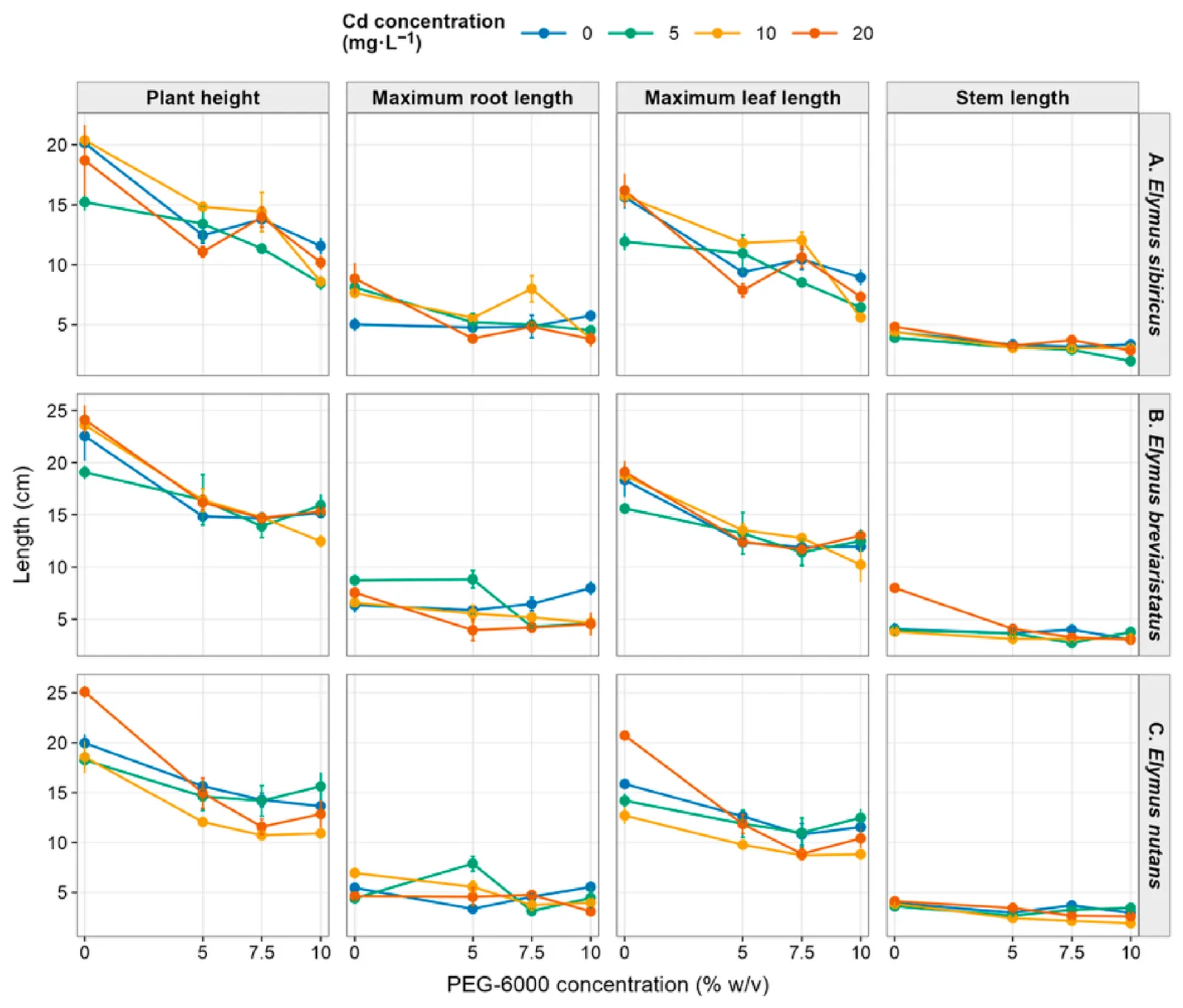
Growth and morphological responses of three forage grasses under combined cadmium and PEG-6000-induced osmotic stress.

**Figure 3:**
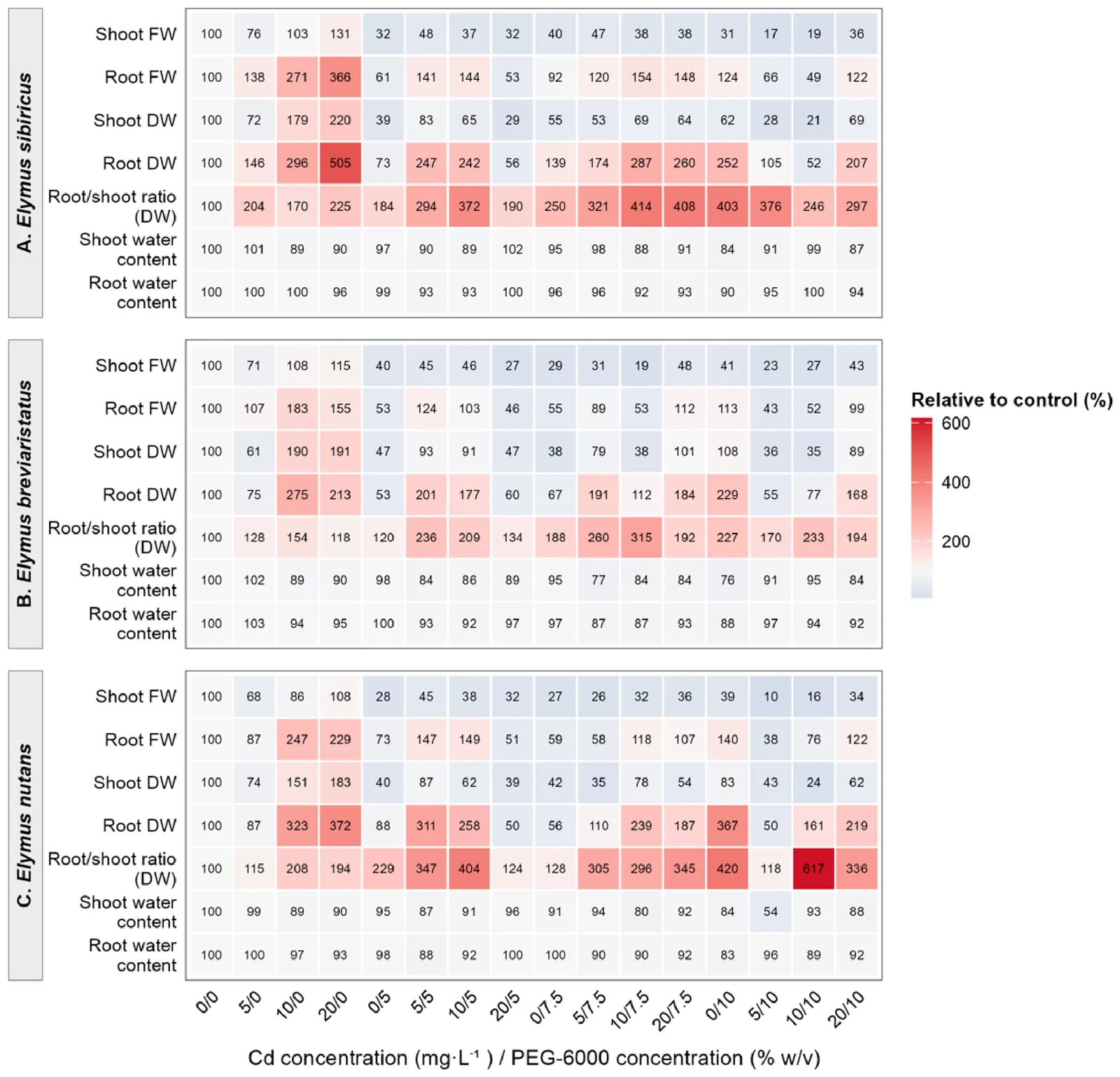
Changes in biomass allocation and water status under combined cadmium and PEG-6000-induced osmotic stress. Note: The heatmaps show shoot fresh weight, root fresh weight, shoot dry weight, root dry weight, root-to-shoot ratio based on dry weight, shoot water content, and root water content under 16 Cd × PEG-6000 treatment combinations. Treatment combinations on the x-axis are presented as Cd concentration (mg·L^−1^)/PEG-6000 concentration (% *w*/*v*). Values are expressed as percentages relative to the species-specific control treatment, defined as 0 mg·L^−1^ Cd + 0% (*w*/*v*) PEG-6000. Values greater than 100% indicate an increase relative to the corresponding control, whereas values below 100% indicate a decrease. Color intensity represents the magnitude of the response relative to the control. Values are means of three biological replicates. FW, fresh weight; DW, dry weight.

**Figure 4:**
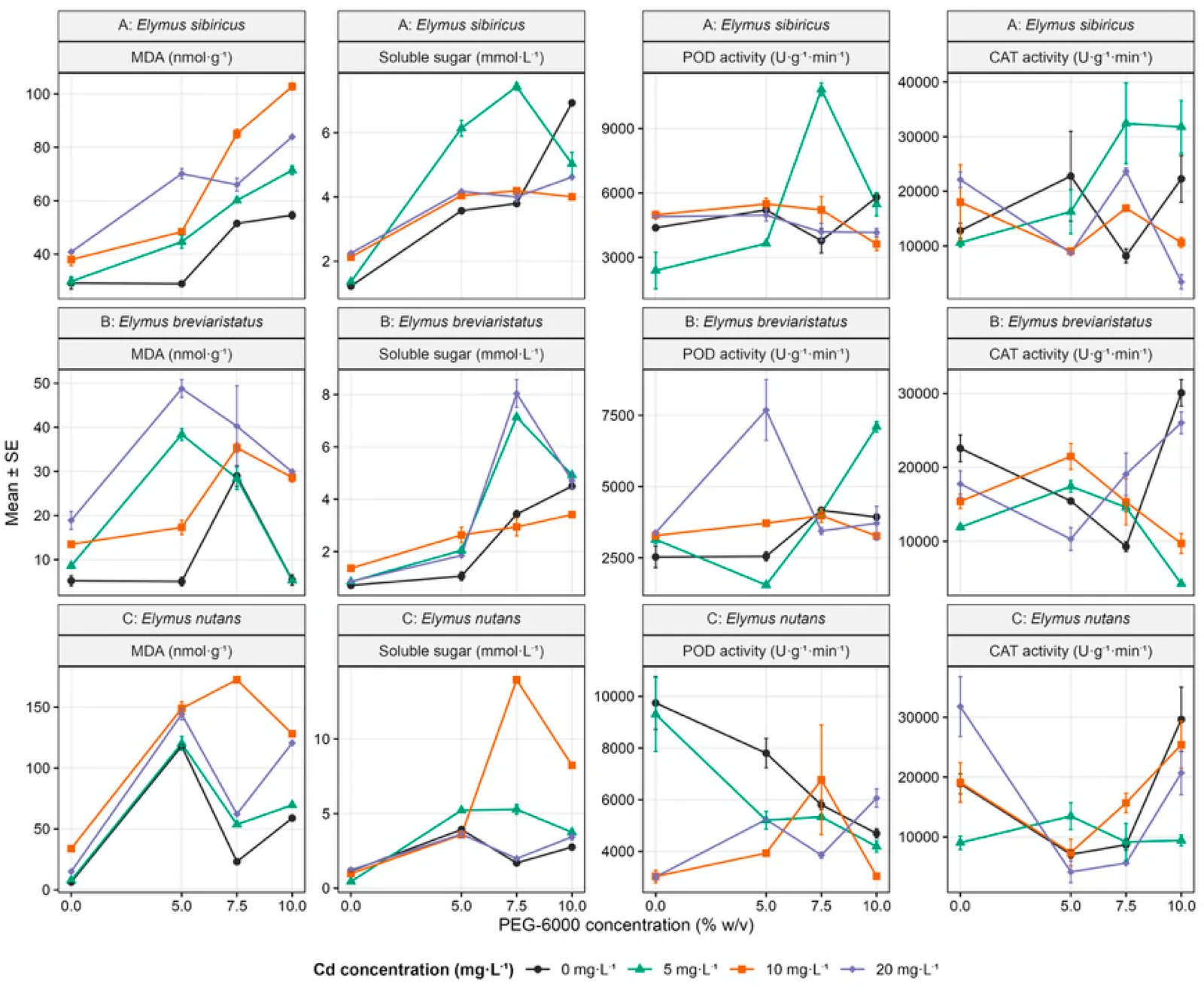
Oxidative damage, osmotic adjustment, and antioxidant enzyme activities under combined cadmium and PEG-6000-induced osmotic stress.

**Figure 5:**
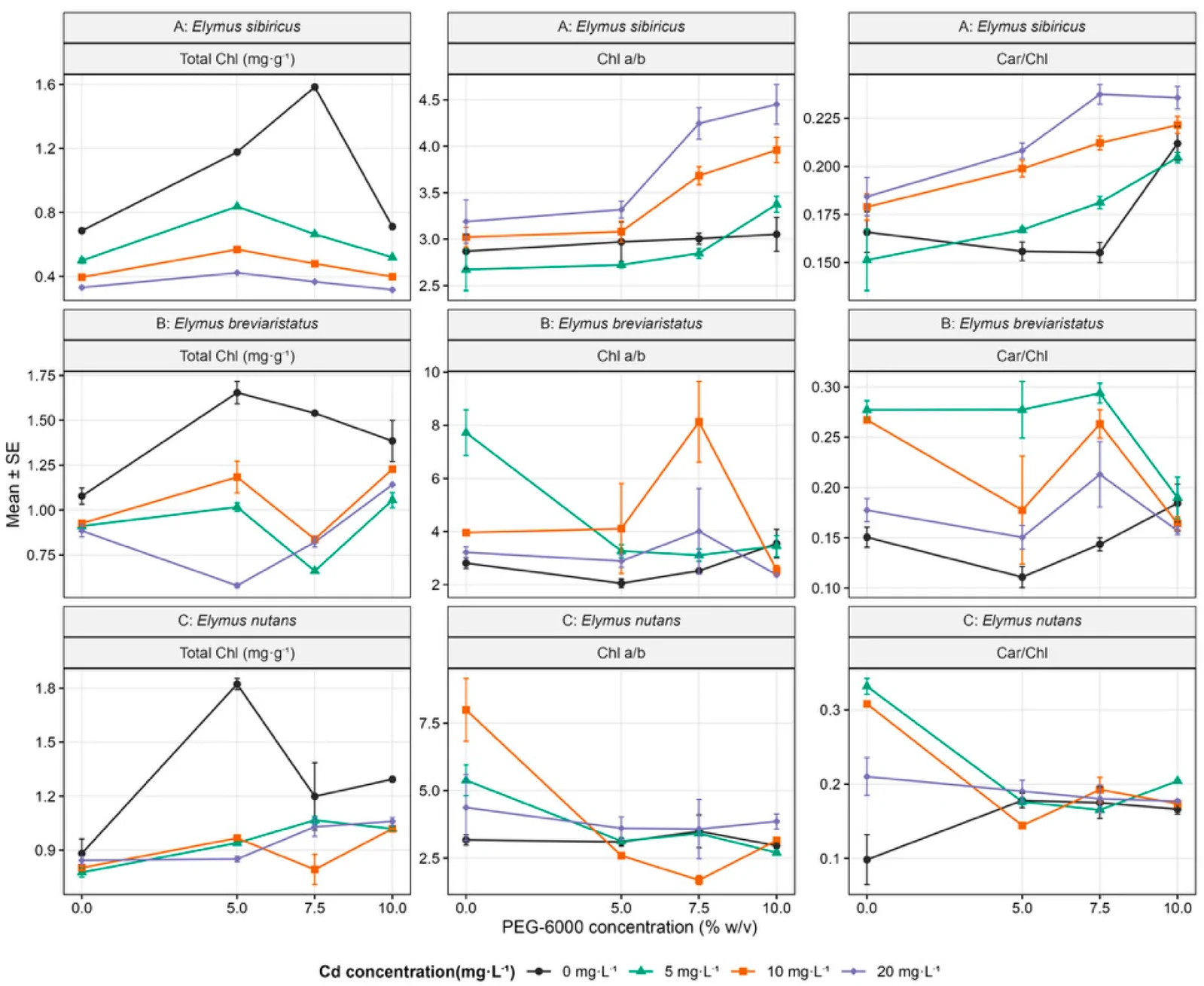
Photosynthetic pigment responses of three forage grasses under combined cadmium and PEG-6000-induced osmotic stress.

Key numerical comparisons under severe combined stress are summarized in Figure 1. The 20 mg·L^−1^ Cd + 10% (*w*/*v*) PEG-6000 treatment clearly distinguished the relative performance of the three grasses. In *E. breviaristatus*, plant height and shoot dry weight were retained at 67.9% and 89.4% of the control, respectively, values higher than or comparable to those of the other two grasses. Its MDA content was 29.91 nmol·g^−1^ FW, significantly lower than those of *E. sibiricus* and *E. nutans*. Meanwhile, *E. breviaristatus* maintained higher CAT activity and total chlorophyll content on a fresh-weight basis. By contrast, *E. sibiricus* showed marked decreases in plant height, CAT activity and total chlorophyll under severe combined stress. *E. nutans* maintained relatively high total chlorophyll and CAT activity but exhibited the highest MDA accumulation. Treatment-response patterns across the Cd × PEG-6000 matrix are presented by trait category in Figure 2, Figure 3, Figure 4 and Figure 5, and complete three-way ANOVA results are provided in Supplementary Table S1.

The results of this section indicate that PEG-6000 is the main factor limiting seedling shoot growth, whereas the Cd background further alters the direction and magnitude of physiological responses under PEG-6000 stress. Differences in measured responses among the three grasses under combined stress were mainly reflected in the degree of coordination among growth maintenance, membrane lipid peroxidation control, antioxidant enzyme activity and photosynthetic pigment stability.

Under the severe combined treatment, *E. breviaristatus* showed comparatively favorable values for several measured traits, including plant height and shoot dry weight retention, absolute MDA content, CAT activity, and total chlorophyll concentration. These observations support a more favorable measured multi-trait response profile under the specific treatment conditions. However, they do not establish whether the lower injury resulted from lower internal Cd exposure or from greater tolerance at a comparable tissue Cd concentration.

### 2.2. Growth and Morphological Responses

Morphological growth of all three grasses generally declined as PEG-6000 concentration increased, but the magnitude of decline differed markedly among species (Figure 2 and Figure S1). Compared with the 0 mg·L^−1^ Cd + 0% (*w*/*v*) PEG-6000 control, plant height of *E. sibiricus*, *E. breviaristatus* and *E. nutans* decreased to 61.9%, 65.8% and 78.4% of their respective controls under 0 mg·L^−1^ Cd + 5% (*w*/*v*) PEG-6000, and to 68.5%, 65.0% and 71.4% under 0 mg·L^−1^ Cd + 7.5% (*w*/*v*) PEG-6000, respectively. Under 0 mg·L^−1^ Cd + 10% (*w*/*v*) PEG-6000, plant height declined to 57.4%, 67.3% and 68.4% of the control in the three grasses, respectively. These results indicate that low-to-moderate PEG-6000-induced osmotic stress was sufficient to markedly restrict seedling shoot elongation. In contrast, the effect of single Cd stress on plant height was more species- and dose-dependent. Under single 5 mg·L^−1^ Cd stress, plant height of the three grasses was 75.7%, 84.6% and 91.5% of the control, respectively, whereas under single 10 mg·L^−1^ Cd stress, it was 101.3%, 104.7% and 93.0%, respectively. Under combined 20 mg·L^−1^ Cd + 10% (*w*/*v*) PEG-6000 stress, plant height further declined to 50.6%, 67.9% and 64.4% of the control, respectively. Height inhibition was most pronounced in *E. sibiricus*, whereas *E. breviaristatus* retained a numerically higher plant-height ratio under severe combined stress.

The sensitivities of root and leaf elongation to combined stress were not completely consistent. Under combined 20 mg·L^−1^ Cd + 10% (*w*/*v*) PEG-6000 stress, maximum root length in *E. sibiricus*, *E. breviaristatus* and *E. nutans* was 3.79, 4.51 and 3.10 cm, respectively, corresponding to 75.5%, 71.1% and 56.8% of their controls. Maximum leaf length was retained at 46.9%, 71.0% and 65.6% of the control, respectively. These results indicate that root elongation of *E. nutans* was more strongly inhibited under severe combined stress, whereas *E. breviaristatus* showed a greater capacity to maintain leaf elongation.

Notably, in the absence of PEG-6000, 20 mg·L^−1^ Cd did not consistently inhibit growth across all species but instead induced dose- and species-dependent growth responses. For example, under 20 mg·L^−1^ Cd + 0% (*w*/*v*) PEG-6000, plant height of *E. breviaristatus* and *E. nutans* increased by 6.9% and 25.6% relative to their controls, respectively, whereas that of *E. sibiricus* decreased by 7.1%. Therefore, single Cd stress and PEG-6000-induced osmotic stress did not affect morphological growth in the same direction, and responses under combined stress should be interpreted in the context of specific Cd × PEG combinations.

### 2.3. Biomass Allocation and Water Status

Biomass allocation showed a clear nonlinear response to Cd × PEG combinations (Figure 3 and Figure S2). Under Cd-free conditions, 5% (*w*/*v*) PEG-6000 reduced shoot fresh weight of *E. sibiricus*, *E. breviaristatus* and *E. nutans* to 32.3%, 40.2% and 28.5% of their controls, respectively; under 7.5% (*w*/*v*) PEG-6000, the corresponding values were 39.9%, 28.6% and 26.9%. These results indicate that shoot fresh weight was highly sensitive to PEG-6000-induced osmotic stress. Under 10 mg·L^−1^ Cd + 7.5% (*w*/*v*) PEG-6000, shoot fresh weight of the three grasses was 37.8%, 19.2% and 32.3% of the control, respectively, indicating that shoot fresh weight remained low when a moderate Cd background was combined with moderate PEG-6000 stress. Under 20 mg·L^−1^ Cd + 10% (*w*/*v*) PEG-6000, shoot dry weight of *E. sibiricus*, *E. breviaristatus* and *E. nutans* was 68.8%, 89.4% and 61.6% of the control, respectively, whereas root dry weight was 207.3%, 168.4% and 219.0% of the control. Compared with shoots, root dry matter was relatively retained or increased under combined stress, suggesting that all three grasses showed some degree of preferential root allocation. *E. breviaristatus*, however, simultaneously maintained a higher level of shoot dry matter.

Water status further indicated that shoots were the more sensitive component under combined stress (Figure 3). Under 20 mg·L^−1^ Cd + 10% (*w*/*v*) PEG-6000, shoot water content of the three grasses was 75.6%, 73.6% and 78.0%, respectively, whereas root water content remained at 84.1%, 82.8% and 82.9%. Three-way ANOVA showed significant Cd × PEG interactions for both shoot water content (*F*_9_,_96_ = 15.79, *p* < 0.001, $\eta_{p}^{2}$ = 0.579) and root water content (*F*_9_,_96_ = 23.52, *p* < 0.001, $\eta_{p}^{2}$ = 0.688), indicating that the effects of PEG-6000 on tissue water status depended on the external Cd concentration. However, these measurements alone do not identify the underlying hydraulic or stomatal processes.

### 2.4. Oxidative Damage, Osmotic Adjustment and Antioxidant Enzyme Systems

MDA content generally increased with increasing combined-stress intensity, indicating that membrane lipid peroxidation was an important physiological response of the three grasses to combined Cd and PEG-6000-induced osmotic stress (Figure 4). Under single PEG-6000 or single Cd treatments, the magnitude of MDA change varied with species and treatment concentration; when high Cd and high PEG-6000 acted together, MDA accumulation was markedly intensified. Under 20 mg·L^−1^ Cd + 10% (*w*/*v*) PEG-6000, MDA contents of *E. sibiricus*, *E. breviaristatus* and *E. nutans* were 83.91, 29.91 and 120.55 nmol·g^−1^ FW, respectively, corresponding to 2.9-, 5.7- and 19.0-fold their respective controls. Based on the additive effects of the single stresses, the theoretical MDA values under 20 mg·L^−1^ Cd + 10% (*w*/*v*) PEG-6000 were approximately 66.23, 19.07 and 67.56 nmol·g^−1^ FW, whereas the measured values were 1.27-, 1.57- and 1.78-fold the theoretical values, respectively, indicating an enhanced lipid peroxidation tendency under high-intensity combined stress. The calculation was performed as follows. For the same grass material, the theoretical additive value under combined stress was calculated as ${MDA}_{add}$ = ${MDA}_{Cd}$ + ${MDA}_{PEG}-{MDA}_{CK}$. Taking the MDA response of *E. sibiricus* under 20 mg·L^−1^ Cd + 10% (*w*/*v*) PEG-6000 as an example, the theoretical additive value calculated from the mean values under single 20 mg·L^−1^ Cd stress, single 10% (*w*/*v*) PEG-6000 stress and the control was 66.23 nmol·g^−1^ FW, whereas the measured value under the combined treatment was 83.91 nmol·g^−1^ FW. Thus, the synergistic damage coefficient was K = 83.91/66.23 = 1.27. This result indicates that measured MDA in *E. sibiricus* under the highest Cd × PEG-6000 combination was approximately 27% higher than the theoretical additive value, suggesting that membrane lipid peroxidation caused by the combined stress was stronger than the simple sum of single-Cd and single-PEG-6000 stress effects. Similarly, the synergistic damage coefficients of *E. breviaristatus* and *E. nutans* were 1.57 and 1.78, respectively, indicating that all three grasses showed different degrees of MDA-enhanced damage tendency under the highest combined stress, with the strongest enhancement in *E. nutans*.

Interspecific comparison showed that although MDA increased relative to the control in *E. breviaristatus* under severe combined stress, its absolute content was the lowest, indicating relatively limited membrane-system damage. *E. nutans* had the highest MDA content and the largest synergistic damage coefficient, indicating greater sensitivity to the combination of high Cd and high PEG-6000. MDA accumulation in *E. sibiricus* was intermediate; however, when combined with its reduced growth and pigment content, this species still displayed clear stress damage. Similar species differentiation was also observed under moderate combined stress. For example, under 10 mg·L^−1^ Cd + 7.5% (*w*/*v*) PEG-6000, MDA contents in the three grasses were 85.09, 35.38 and 172.49 nmol·g^−1^ FW, respectively; *E. breviaristatus* still maintained a lower level, whereas oxidative damage was most severe in *E. nutans*.

Soluble sugars were sensitive to PEG-6000-induced osmotic stress (Figure 4). Under 0 mg·L^−1^ Cd + 10% (*w*/*v*) PEG-6000, soluble sugar contents of *E. sibiricus*, *E. breviaristatus* and *E. nutans* increased from control values of 1.23, 0.71 and 1.15 mmol·L^−1^ to 6.93, 4.50 and 2.75 mmol·L^−1^, respectively, indicating that PEG-6000 stress induced osmotic adjustment responses in all three grasses. Under moderate combined stress, interspecific differences became more pronounced. Under 10 mg·L^−1^ Cd + 7.5% (*w*/*v*) PEG-6000, soluble sugar contents were 4.19, 2.94 and 13.97 mmol·L^−1^, respectively. *E. nutans* accumulated the highest amount of soluble sugars, but its MDA content was also high, indicating that extensive soluble sugar accumulation did not effectively alleviate membrane lipid peroxidation damage in this species.

Under the highest combined stress, the high-Cd background affected PEG-6000-induced sugar accumulation in a clearly species-specific manner. Under 20 mg·L^−1^ Cd + 10% (*w*/*v*) PEG-6000, soluble sugar content in *E. sibiricus* was 4.62 mmol·L^−1^, lower than its level under single 10% (*w*/*v*) PEG-6000 stress. In *E. breviaristatus* and *E. nutans*, soluble sugar contents were 4.72 and 3.43 mmol·L^−1^, respectively, both higher than their respective levels under single 10% (*w*/*v*) PEG-6000 stress. Together with the MDA results, *E. breviaristatus* showed relative coordination among soluble sugar maintenance, lower MDA and better shoot growth maintenance. Although *E. nutans* accumulated soluble sugars substantially under some treatments, membrane lipid peroxidation damage remained severe.

Antioxidant enzyme activities showed strong treatment dependence, and CAT more clearly reflected differences in oxidative defense among grasses under severe combined stress (Figure 4). Under 20 mg·L^−1^ Cd + 10% (*w*/*v*) PEG-6000, CAT activity in *E. sibiricus* decreased from 12,800 U·g^−1^ FW·min^−1^ in the control to 3400 U·g^−1^ FW·min^−1^, whereas *E. breviaristatus* and *E. nutans* maintained CAT activities of 26,025 and 20,700 U·g^−1^ FW·min^−1^, respectively. Simple-effect comparison among species showed that CAT activity in both *E. breviaristatus* and *E. nutans* was significantly higher than that in *E. sibiricus*, while the difference between *E. breviaristatus* and *E. nutans* was not significant. When combined with MDA content, the higher CAT activity of *E. breviaristatus* was consistent with its lower MDA level. In contrast, *E. nutans* maintained high CAT activity but still accumulated large amounts of MDA, indicating a partial decoupling between antioxidant enzyme maintenance and membrane damage control.

These results indicate that all three grasses underwent changes in membrane lipid peroxidation, osmotic adjustment and antioxidant enzyme activity under combined Cd and PEG-6000-induced osmotic stress, but tolerance did not depend on the increase of any single trait. The measured response of *E. breviaristatus* was characterized by the co-occurrence of lower absolute MDA content, maintained CAT activity, relatively stable soluble sugar levels, and better growth retention. *E. nutans* showed strong sugar and CAT responses but severe membrane lipid peroxidation, whereas *E. sibiricus* showed reduced CAT activity and clear oxidative damage under severe combined stress.

### 2.5. Photosynthetic Pigment Responses

Total chlorophyll content on a fresh-weight basis was interactively regulated by grass species, Cd and PEG-6000, with a highly significant three-way interaction (Grass × Cd × PEG: *F*_18_, _96_ = 11.21, *p* < 0.001, $\eta_{p}^{2}$ = 0.678) (Figure 5 and Figure S3). This indicates clear species-specific responses of photosynthetic pigments to combined Cd-PEG stress. Overall, under low-to-moderate stress gradients, total chlorophyll content per unit fresh weight was not fully consistent with biomass changes. Under 10 mg·L^−1^ Cd + 5% (*w*/*v*) PEG-6000, total chlorophyll in *E. breviaristatus* was 109.8% of the control, but it decreased to 77.7% under 10 mg·L^−1^ Cd + 7.5% (*w*/*v*) PEG-6000. In *E. nutans*, total chlorophyll remained at 120.9% of the control under 5 mg·L^−1^ Cd + 7.5% (*w*/*v*) PEG-6000, whereas MDA was also at a high level. Therefore, pigment traits should be interpreted together with fresh weight, biomass and oxidative damage. Under 20 mg·L^−1^ Cd + 10% (*w*/*v*) PEG-6000, total chlorophyll on a fresh-weight basis differed markedly among the three grasses. In *E. sibiricus*, total chlorophyll decreased from 0.686 mg·g^−1^ FW to 0.317 mg·g^−1^ FW, only 46.2% of the control. In *E. breviaristatus* and *E. nutans*, total chlorophyll contents were 105.9% and 120.2% of their controls, respectively. Simple-effect comparison among species showed that total chlorophyll contents of *E. breviaristatus* and *E. nutans* were both significantly higher than that of *E. sibiricus*, whereas the difference between the former two species was not significant. This indicates that *E. breviaristatus* and *E. nutans* had a stronger capacity to maintain pigment concentration on a fresh-weight basis under severe combined stress.

It should be noted that an increase in total chlorophyll content on a fresh-weight basis does not necessarily indicate an increase in total shoot pigment accumulation. After conversion using shoot fresh weight, under 20 mg·L^−1^ Cd + 10% (*w*/*v*) PEG-6000, shoot fresh weight of *E. sibiricus*, *E. breviaristatus* and *E. nutans* was 36.4%, 42.8% and 33.6% of the control, respectively. When shoot total chlorophyll amount was calculated from raw replicate data, the relative shoot total chlorophyll amounts under severe combined stress were only 16.9%, 45.1% and 39.7% of the control, respectively. These results indicate that although total chlorophyll per unit fresh weight in *E. breviaristatus* and *E. nutans* was higher than or close to the control level, their total shoot chlorophyll amount remained substantially lower than the control because shoot fresh weight declined markedly. Therefore, the increase in total chlorophyll on a fresh-weight basis under severe combined stress should be interpreted as pigment maintenance or concentration changes per unit fresh weight, potentially including concentration effects caused by reduced tissue water content and growth inhibition, rather than as evidence that Cd-PEG combined stress promoted whole-plant photosynthetic pigment accumulation or enhanced photosynthetic capacity.

From the perspective of interspecific differences, *E. breviaristatus* simultaneously showed a higher level of total chlorophyll maintenance on a fresh-weight basis, a higher estimated relative amount of shoot total chlorophyll, and lower MDA content under severe combined stress, indicating good consistency between pigment maintenance and membrane lipid peroxidation control. Although *E. nutans* had the highest relative total chlorophyll value per unit fresh weight, its shoot fresh weight declined markedly, and the converted relative amount of shoot total chlorophyll was lower than that of *E. breviaristatus*; meanwhile, it had the highest MDA accumulation. This indicates that pigment maintenance on a fresh-weight basis in *E. nutans* did not translate into lower oxidative damage. *E. sibiricus* showed simultaneous decreases in total chlorophyll per unit fresh weight, shoot fresh weight and estimated relative shoot total chlorophyll amount, indicating that its photosynthetic pigment system was more strongly affected by severe combined stress.

In addition, Chl a/b in *E. sibiricus* increased to 4.45 and carotenoid (Car)/total Chl increased to 0.236 under 20 mg·L^−1^ Cd + 10% (*w*/*v*) PEG-6000. However, because both total chlorophyll per unit fresh weight and estimated relative shoot total chlorophyll amount decreased markedly, these ratio changes are more likely to reflect stress-induced relative restructuring of pigment components and should not be interpreted alone as improved photosynthetic performance. Overall, *E. breviaristatus* showed relatively better pigment maintenance under severe combined stress, but the converted relative amount of shoot total chlorophyll in all three grasses was lower than the control, indicating that combined stress still weakened shoot photosynthetic pigment accumulation.

### 2.6. Integrated Multi-Trait Response Evaluation Based on PCA, Stress Tolerance Coefficient, and Membership Function Analysis

**Figure 6:**
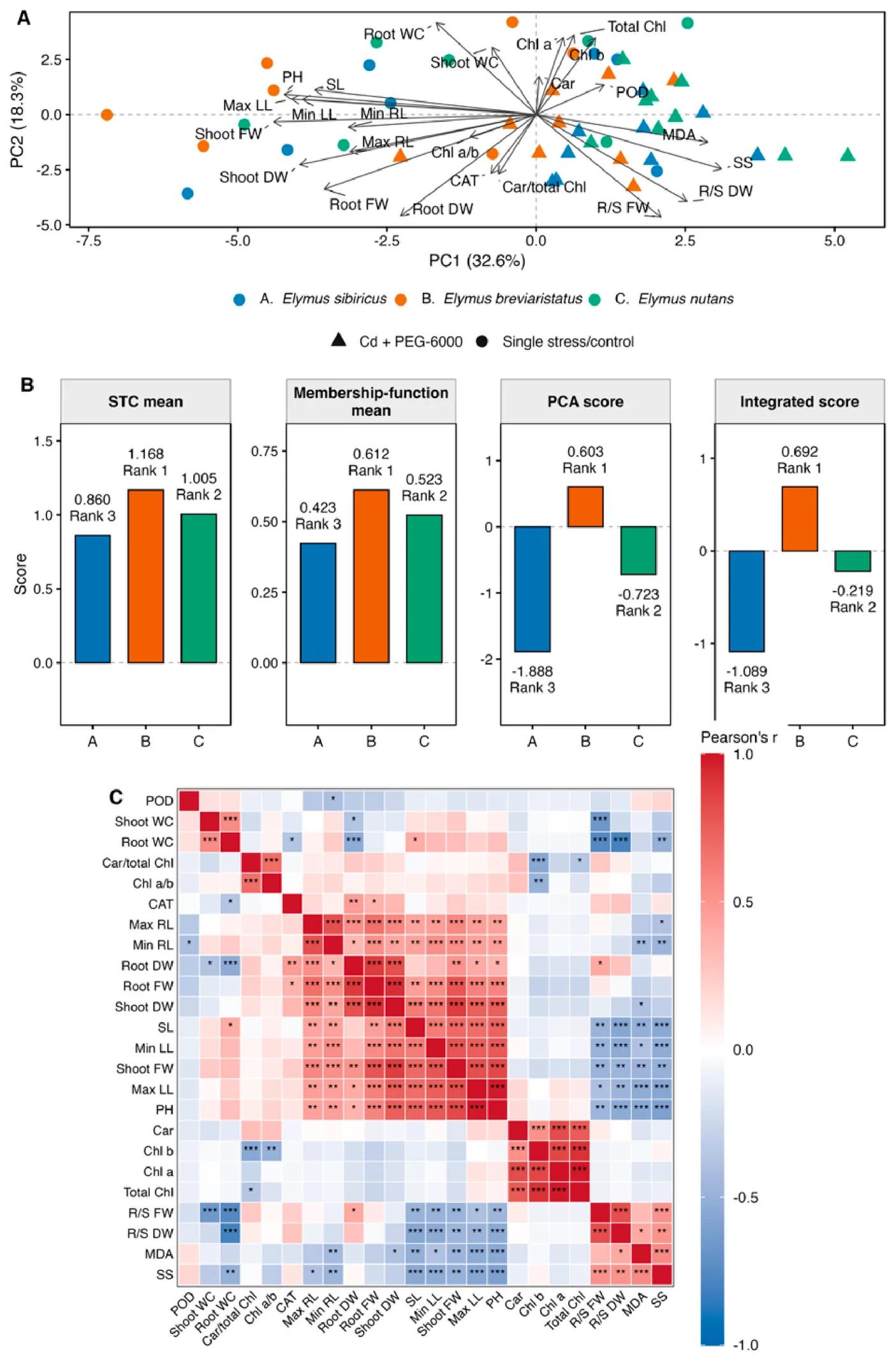
Integrated multivariate evaluation of Cd–PEG-6000-induced osmotic-stress responses in three *Elymus* grasses. (**A**) PCA biplot based on treatment means and standardized growth, biomass, water-status, physiological, and pigment traits. Colors represent *Elymus sibiricus*, *E. breviaristatus*, and *E. nutans*. Triangles represent combined Cd + PEG-6000 treatments, whereas circles represent single-stress or control treatments. (**B**) Comparison of the STC mean, membership function mean, PCA score, and integrated score under the severe combined treatment of 20 mg·L^−1^ Cd and 10% (*w*/*v*) PEG-6000. A, B, and C represent *E. sibiricus*, *E. breviaristatus*, and *E. nutans*, respectively. Values and corresponding ranks are shown adjacent to the bars. Because the four indices have different numerical scales, they are presented in separate panels with independent y-axes. (**C**) Pearson correlation heatmap among the measured traits. Red and blue indicate positive and negative correlations, respectively. Asterisks indicate significance levels: * *p* < 0.05, ** *p* < 0.01, and *** *p* < 0.001.

PCA based on 48 treatment means and 20 standardized traits showed that principal component 1 (PC1) and principal component 2 (PC2) explained 32.6% and 18.3% of the total variation, respectively, with a cumulative explanation of 50.9% (Figure 6A). PC1 mainly reflected integrated differences among growth maintenance, biomass accumulation and stress damage. In the PCA biplot, growth and biomass traits, including plant height, stem length, leaf length, shoot fresh weight and shoot dry weight, generally clustered in the same direction, whereas MDA, soluble sugars and root/shoot ratio were in the opposite direction. This indicates a clear association among growth inhibition, oxidative damage and biomass allocation adjustment under combined stress. PC2 was mainly related to chlorophyll content, tissue water content and some antioxidant enzyme activities, reflecting differences in pigment maintenance, water status and physiological defense.

The cumulative explanation of PC1 and PC2 was 50.9%, indicating that the two-dimensional PCA plot was mainly suitable for displaying multivariate distribution patterns among treatments and should not be used alone for tolerance ranking. Therefore, this study further combined stress tolerance coefficient (STC), membership function analysis and PCA integrated scores based on the first five principal components for cross-validation. STC analysis used the species-specific control as the baseline and reflected the maintenance capacity of each trait under different stress treatments relative to the control. Membership function analysis further compared the relative performance of the three grasses for each trait within the same Cd × PEG combination. The PCA integrated score was used to summarize the overall response pattern reflected by coordinated multi-trait variation. Under severe combined 20 mg·L^−1^ Cd + 10% (*w*/*v*) PEG-6000 stress, STC, membership function analysis and PCA integrated scores all ranked *E. breviaristatus* first, with STC mean, membership mean, PCA score and integrated score of 1.168, 0.612, 0.603 and 0.692, respectively (Figure 6B and Table 1). *E. nutans* ranked second overall, with an integrated score of −0.219, whereas *E. sibiricus* ranked third, with an integrated score of −1.089. This result was generally consistent with individual traits: *E. breviaristatus* maintained higher plant height and shoot dry weight retention, lower absolute MDA content, and better maintenance of CAT activity and total chlorophyll on a fresh-weight basis under severe combined stress. By contrast, *E. sibiricus* showed more pronounced shoot growth inhibition, reduced CAT activity and lower total chlorophyll, whereas *E. nutans* performed well for some water and pigment traits but accumulated high MDA, indicating severe membrane lipid peroxidation damage. Overall, the tolerance assessment for *E. breviaristatus* was not derived from PCA alone but was jointly supported by STC, membership function analysis, PCA integrated scores and key individual physiological traits. Because integrated tolerance evaluation depends on trait selection, direction correction and standardization methods, this study did not use PCA or any single integrated score as the sole criterion; instead, these indices were interpreted together with individual traits such as growth, biomass, MDA, antioxidant enzyme activities and photosynthetic pigments. Pearson correlation analysis further showed that most growth and biomass traits were positively correlated with one another but negatively correlated with MDA and soluble sugars, while chlorophyll a, chlorophyll b, and total chlorophyll formed a strongly positively correlated group (Figure 6C).

## 3. Discussion

### 3.1. Cd and PEG-6000 Combined Stress Induces Trait-Dependent Non-Additive Responses

This study showed that Cd and PEG-6000-induced osmotic stress jointly altered growth, biomass allocation, water status, oxidative damage, osmotic adjustment, antioxidant enzyme activities and photosynthetic pigments in three alpine Elymus grasses. The significant Cd × PEG and Grass × Cd × PEG interactions indicate that combined-stress responses were not a simple superposition of the two single stresses. Previous studies have suggested that multiple stresses are co-regulated by interacting or even antagonistic signaling pathways, so their combined effects may be synergistic, antagonistic or additive [11]. The present results further show that this non-additivity was strongly trait- and species-dependent.

Membrane lipid peroxidation showed the clearest amplification under severe combined stress: measured MDA exceeded the theoretical additive response in all three grasses, indicating convergence between Cd-induced ROS pressure and osmotic-stress-related disruption of redox homeostasis. Cd can inhibit plant growth by disrupting mineral nutrition, damaging photosynthetic structures and promoting ROS accumulation, whereas water deficit further restricts cell elongation, carbon assimilation and antioxidant homeostasis [12,13,14]. However, combined stress did not uniformly produce synergistic aggravation for all traits. PEG-6000 was the principal limitation on shoot growth, while Cd modified the magnitude and direction of growth and biomass responses in a dose- and species-dependent manner. The relative maintenance of root dry matter, together with reduced shoot fresh weight and whole-shoot pigment accumulation, further indicates that above- and belowground responses were not equivalent. Thus, the non-additive effect should be interpreted as a trait-dependent interaction across physiological levels rather than as generalized synergistic injury.

Most previous *Elymus* studies have focused on PEG-6000-induced osmotic stress or Cd stress separately. PEG-6000 can induce differential responses in *E. nutans* and *E. sibiricus*, with more favorable materials generally maintaining higher relative water content and antioxidant activity and lower oxidative damage [15], while Cd affects growth, antioxidant, and MDA or proline accumulation in *E. dahuricus* [16]. Comparative information under combined Cd and PEG-6000 stress remains limited. The contribution of the present study is therefore to characterize contrasting multi-trait response profiles among three alpine perennial *Elymus* grasses within the same factorial system.

Accordingly, the responses of alpine forage grasses to combined Cd and PEG-6000-induced osmotic stress should be evaluated by integrating growth maintenance, membrane lipid peroxidation, osmotic adjustment, antioxidant defense and pigment stability rather than by relying on any single indicator.

### 3.2. Growth Inhibition Is Closely Related to Biomass Allocation and Water Status Changes

PEG-6000 progressively inhibited shoot growth in all three grasses, but the magnitude differed among species. *E. breviaristatus* maintained comparatively better plant height and shoot-biomass retention under severe combined stress, whereas shoot growth declined more strongly in *E. sibiricus* and *E. nutans*. This contrast indicates that maintenance of aboveground growth was an important component of favorable relative performance.

All three grasses relatively maintained or increased root dry matter under severe combined stress, suggesting a shift in allocation toward belowground tissues. Such allocation is a common response to water limitation and may support water acquisition [17,18], but root dry matter alone does not demonstrate improved water uptake or hydraulic conductance. *E. nutans* maintained high root biomass but showed the strongest MDA accumulation, indicating that root allocation did not prevent physiological injury. In contrast, *E. breviaristatus* combined better shoot retention with lower MDA and more stable pigment traits. Shoot water content also declined more strongly than root water content, identifying aboveground tissues as the more responsive component. Nevertheless, tissue water content alone cannot identify the processes underlying the significant Cd × PEG interaction.

One biologically plausible explanation is that Cd toxicity and PEG-6000-induced osmotic stress converged on the plant water-transport continuum. PEG-6000 lowered external water potential and restricted root water uptake, while previous studies have shown that Cd can increase stomatal resistance, inhibit stomatal opening, reduce stomatal conductance and transpiration, and modify root-to-shoot hydraulic transport [19,20,21,22,23]. Their interaction could therefore contribute to growth inhibition through hydraulic-stomatal limitation as well as oxidative injury.

This interpretation remains hypothetical because stomatal conductance, transpiration rate, leaf water potential, root and leaf hydraulic conductance, xylem sap flow, and aquaporin activity were not measured. Consequently, the observed Cd × PEG interaction in tissue water status should be interpreted as a treatment-dependent water-status response rather than as direct evidence of Cd-induced stomatal closure or hydraulic impairment. Future experiments integrating water-relation measurements with oxidative-damage indicators are needed to distinguish hydraulic–stomatal limitation from ROS-associated injury.

### 3.3. Coupling Among Oxidative Damage, Osmotic Adjustment and Antioxidant Defense

MDA accumulation confirmed that membrane lipid peroxidation was a central component of the combined-stress response, but damage intensity differed strongly among species. MDA should therefore be interpreted together with antioxidant activity and growth performance [24,25]. *E. breviaristatus* had the lowest absolute MDA content under severe combined stress and retained comparatively better growth, indicating more effective membrane protection. *E. nutans* showed the strongest lipid peroxidation despite maintaining some defensive traits, whereas *E. sibiricus* combined oxidative injury with reduced CAT activity, growth inhibition and chlorophyll loss. These differences indicate that membrane protection, rather than the activation of any single defense trait, was critical to relative performance.

Soluble sugars accumulated mainly in response to PEG-6000, consistent with their functions in osmotic adjustment, carbon storage, ROS scavenging and stress signaling [26]. A high-Cd background, however, modified this response in a species-specific manner. In *E. sibiricus*, severe Cd exposure appeared to suppress PEG-induced sugar accumulation, whereas *E. breviaristatus* and *E. nutans* maintained an osmotic-adjustment response. Importantly, high sugar accumulation in *E. nutans* did not prevent severe membrane lipid peroxidation. By contrast, *E. breviaristatus* showed closer coordination among soluble-sugar maintenance, lower MDA and better growth retention. Thus, osmolyte accumulation alone was not a reliable indicator of comprehensive performance.

### 3.4. The Antioxidant Enzyme System Shows Strong Species and Treatment Dependence

The antioxidant enzyme system also responded in a strongly species-specific manner. CAT activity declined in *E. sibiricus* under severe combined stress, which was consistent with weakened ROS-scavenging capacity, chlorophyll loss, oxidative damage and growth inhibition [24]. By contrast, *E. breviaristatus* and *E. nutans* maintained relatively high CAT activity.

However, higher antioxidant enzyme activity does not necessarily indicate stronger comprehensive tolerance. *E. nutans* maintained high CAT activity and chlorophyll concentration but also accumulated the highest MDA, revealing a clear mismatch between CAT maintenance and membrane protection. CAT removes H2O2, whereas effective ROS detoxification requires coordinated enzymatic and non-enzymatic components, including SOD, POD, ascorbate peroxidase (APX), glutathione reductase (GR) and the ascorbic acid-glutathione (AsA-GSH) cycle [2]. High CAT activity alone therefore cannot guarantee control of all ROS or prevent oxidative damage. The observed CAT-MDA mismatch could reflect incomplete coordination among defense pathways, differences in Cd uptake, root retention or shoot translocation, or differences in tissue sensitivity to internal Cd. Because Cd availability, tissue concentrations, translocation, subcellular distribution and cell-wall immobilization were not measured, these alternatives cannot be distinguished. The pattern is therefore interpreted as a measured response profile rather than evidence of a specific Cd-transport or tolerance mechanism [27].

### 3.5. Photosynthetic Pigment Responses Under Combined Stress

Photosynthetic pigments showed marked interspecific differences under combined stress. Cd can reduce photosynthetic capacity by damaging chlorophyll structure, interfering with pigment synthesis and disrupting electron transport, while water limitation can further destabilize the photosynthetic apparatus [28,29,30]. In *E. sibiricus*, the simultaneous decline in total chlorophyll and shoot biomass indicates strong pigment-system sensitivity. The increases in Chl a/b and Car/total Chl should be interpreted cautiously because they occurred while both pigment concentration and estimated whole-shoot pigment amount declined; these ratio changes more likely reflect stress-induced restructuring of pigment composition than improved photosynthetic performance.

By contrast, *E. breviaristatus* and *E. nutans* maintained relatively high chlorophyll concentration on a fresh-weight basis under severe combined stress. Chlorophyll responses are strongly dependent on genotype and stress intensity [31,32], and stronger antioxidant protection may help stabilize chloroplast membranes and pigment molecules [24,33]. However, chlorophyll concentration was expressed per unit fresh weight, so reduced leaf growth and tissue water content may also have produced a concentration effect. After accounting for the marked loss of shoot fresh weight, whole-shoot chlorophyll accumulation remained below the control in all three grasses. Therefore, pigment concentration should not be equated with enhanced whole-plant photosynthetic capacity. *E. breviaristatus* combined pigment maintenance with lower MDA and better biomass retention, whereas the pigment stability of *E. nutans* did not prevent severe oxidative damage. Gas-exchange and chlorophyll-fluorescence measurements are required to determine whether pigment maintenance translated into functional photosynthetic performance.

### 3.6. Contrasting Measured Response Profiles and Integrated Evaluation of the Three Grasses

Based on the measured traits, the three grasses exhibited contrasting response profiles. *E. sibiricus* showed pronounced reductions in shoot growth, CAT activity, and chlorophyll traits and was characterized as growth- and pigment-sensitive. *E. nutans* maintained relatively high CAT activity and chlorophyll concentration but accumulated high MDA and was characterized as having an antioxidant/pigment-maintenance–damage-decoupled response profile. *E. breviaristatus* simultaneously showed better growth retention, lower absolute MDA content, maintained CAT activity, and comparatively favorable pigment traits and was therefore characterized as having a coordinated measured multi-trait response profile. These descriptors summarize the observed phenotypic and physiological traits only. They do not establish differences in Cd exclusion, root retention, root-to-shoot translocation, internal Cd exposure, or tissue-level Cd tolerance.

PCA and integrated response indices supported the cross-trait interpretation of the three grasses. The PCA pattern linked growth and biomass maintenance with oxidative damage and allocation adjustment, while the correlation analysis supported the inclusion of both growth maintenance and membrane protection in the integrated evaluation. These multivariate tools therefore complemented, rather than replaced, the interpretation of individual physiological traits.

Multi-method evaluation consistently supported the comparatively favorable response profile of *E. breviaristatus*, which ranked first in the STC, membership-function, PCA-based and integrated assessments under severe combined stress. The importance of this agreement lies less in the numerical scores than in their consistency with the individual traits: E. breviaristatus combined better shoot retention, lower MDA, and maintained CAT and pigment traits; *E. nutans* retained some water and pigment traits but experienced severe membrane damage; and *E. sibiricus* showed stronger growth inhibition, lower CAT and chlorophyll loss. *E. breviaristatus* should therefore be considered a candidate for further validation under the tested conditions. The corresponding score comparison and exact numerical values are presented in Figure 6B and Table 1, respectively.

It should be noted that PCA-based integrated evaluation is essentially a dimensionality-reduction analysis based on correlation structure and variance contribution. Its results depend on the included traits, standardization method and score-direction settings. Therefore, this study did not use PCA score alone as the criterion for tolerance assessment; instead, PCA was interpreted together with individual traits, including growth, biomass, MDA, antioxidant enzyme activities and photosynthetic pigments. Importantly, the integrated ranking reflects relative performance under a common nominal external Cd treatment. It does not represent a comparison of tolerance at an equivalent internal Cd concentration, because internal Cd exposure was not quantified.

### 3.7. Ecological Significance and Study Limitations

Within the controlled hydroponic system, *E. breviaristatus* showed the most favorable coordinated response and can therefore be considered a candidate for further mechanistic and soil-based validation. This conclusion is restricted to relative multi-trait performance at the seedling stage and should not be extrapolated directly to intrinsic Cd tolerance, forage safety, phytoremediation potential or long-term field performance. Because no site-specific soil Cd concentrations, soil Cd availability, or soil-water status were measured, the actual Cd exposure and drought intensity experienced by these materials under cultivation could not be quantified in the present study.

The most important limitation is the absence of information on Cd availability and internal exposure. Although nominal external concentrations were controlled, actual solution Cd availability, root and shoot Cd concentrations and contents, bioconcentration, translocation, subcellular distribution, cell-wall immobilization and chemical forms were not quantified. Consequently, the favorable response of *E. breviaristatus* could reflect lower uptake, stronger exclusion, greater root retention, reduced translocation, greater tolerance at comparable internal Cd or a combination of these processes [27,34,35,36]. The high MDA of *E. nutans* likewise cannot be assigned to a particular Cd-uptake or distribution mechanism. A second limitation is the use of a 28-day hydroponic seedling system. Although this design reduced variation associated with soil heterogeneity and enabled standardized interspecific comparison, it did not reproduce key features of natural soil conditions, including heterogeneous water distribution, root–soil hydraulic resistance, nutrient mobility, aeration, rhizosphere physicochemical processes, microbial interactions, temporal fluctuations in water availability, or the long-term behavior of perennial plants [37,38]. These differences may also affect Cd speciation and bioavailability and therefore limit direct extrapolation of the present findings to natural or semi-natural environments.

A third limitation concerns the range of measurements. The study did not assess several important antioxidant pathways, non-enzymatic defenses, root architecture and activity, leaf water potential, stomatal hydraulic traits, gas exchange or chlorophyll fluorescence. Consequently, the significant water-status interaction cannot be attributed specifically to stomatal closure, restricted transpiration, impaired root water uptake or altered hydraulic transport [19,20,21,22,23]. The observed MDA, enzyme, water-status and pigment responses therefore describe associated physiological patterns but do not establish complete causal pathways [24,28,29,33].

Future research should proceed in three stages. First, controlled mechanistic experiments should quantify Cd availability, root and shoot Cd accumulation, bioconcentration, translocation and subcellular distribution. These measurements should be integrated with leaf water potential, stomatal conductance, transpiration, gas exchange, hydraulic conductance, aquaporin activity, antioxidant pathways and root traits to distinguish Cd partitioning, hydraulic-stomatal limitation and tissue-level tolerance.

Second, factorial soil-pot experiments should test whether the relative ranking persists under realistic soil Cd availability and water supply. Measurements should include rhizosphere Cd, root and shoot accumulation, soil physicochemical properties, root traits, microbial communities, plant water relations and biomass production.

Third, multi-year field or field-plot trials should evaluate establishment, overwintering, regrowth, adult-plant biomass, persistence, competitive ability and reproduction under alpine conditions. Cd accumulation, forage safety, food-chain transfer risk, soil Cd availability and effects on surrounding plant and microbial communities should also be assessed. Only through this sequential validation can the field applicability of the present findings be evaluated reliably.

## 4. Materials and Methods

### 4.1. Plant Materials and Seedling Culture

Three Poaceae forage grasses were used as experimental materials: *Elymus sibiricus* cv. Chuancao, *Elymus breviaristatus* and *Elymus nutans* Griseb. cv. Gannan. The seeds were supplied by the Tongde Forage Seed Breeding Farm in Qinghai Province and were maintained and propagated by the same breeding unit. The seeds were harvested in 2025 and stored at 4 °C under dry and dark conditions before the experiment. Plump, uniform, undamaged and mold-free seeds were selected, surface-sterilized with 2% sodium hypochlorite for 15 min, repeatedly rinsed with ultrapure water until no residue remained, and then air-dried. The sterilized seeds were evenly sown in seedling trays and germinated in an illuminated growth chamber for 7 d. Before transplantation, oversized, undersized and abnormal seedlings were removed, and 7-day-old seedlings with uniform age, intact root systems and similar growth status were selected for transplanting. Hydroponic culture was performed in sterilized Petri dishes with a diameter of 9 cm. Each dish received 10 mL of the corresponding Cd × PEG-6000 treatment solution and was planted with 30 seedlings; each dish was regarded as one biological replicate, and each treatment included three biological replicates. The Petri dishes were randomly arranged in the growth chamber and periodically repositioned during stress treatment to minimize microenvironmental differences in light and temperature. The growth conditions were set to 14 h light at 25 °C and 10 h dark at 20 °C. The corresponding fresh treatment solution was replaced every 2 d, and samples were collected for all measurements after 28 d of continuous stress treatment. This indoor hydroponic seedling system was used to reduce interference from soil heterogeneity on Cd and PEG-6000 treatment effects and to compare the relative physiological responses of the three grasses under uniform controlled conditions.

### 4.2. Combined Cd and PEG-6000 Stress Treatments

All treatment solutions were prepared using commercial Hoagland nutrient solution as the basal medium. The Hoagland solution consisted of three concentrated components (A, B and C) purchased from Xiqingchun Agricultural Technology. Before use, the A, B and C concentrates were diluted and mixed according to the manufacturer’s instructions to prepare 1× Hoagland working solution. Cd stress was imposed using CdCl_2_·2.5H_2_O as the Cd source at four levels: 0 (CK), 5, 10 and 20 mg·L^−1^ (all Cd concentrations in this study refer to the elemental Cd mass concentration). PEG-6000 was applied at four concentrations: 0% (CK), 5%, 7.5%, and 10% (*w*/*v*). The treatment solutions were prepared by adding the corresponding Cd solution and PEG-6000 to the Hoagland working solution to reach the target final concentrations, yielding 16 Cd × PEG treatment combinations. The nominal Cd concentrations were selected with reference to a previous 28 d hydroponic study of *Elymus nutans*, in which 0, 10, 20, and 40 mg·L^−1^ Cd were applied [39]. In the present study, the upper Cd concentration was limited to 20 mg·L^−1^, and an additional level of 5 mg·L−1 was included to establish a graded four-level external-exposure series suitable for full-factorial analysis. The PEG-6000 concentrations of 5%, 7.5%, and 10% (*w*/*v*) were selected to generate progressively stronger osmotic-stress levels. Based on the empirical relationship reported by Michel and Kaufmann [40], these concentrations correspond to estimated osmotic potentials of approximately −0.050, −0.093, and −0.148 MPa at 25 °C, respectively. Together with the corresponding zero controls, these treatment levels provided a graded 4 × 4 matrix for evaluating concentration-dependent and interactive Cd–PEG effects under standardized hydroponic conditions.

From a physiological perspective, the selected concentration ranges were intended to establish progressively increasing stress intensities and to enable the detection of concentration-dependent changes in growth, tissue water status, osmotic adjustment, oxidative damage, antioxidant activity, and photosynthetic pigments, while retaining sufficient viable plant material for interspecific comparison. The PEG-6000 gradient represented progressively greater reductions in external water potential and was used to impose increasing osmotic-stress intensity under controlled conditions [5,40]. Similarly, the Cd gradient was designed to generate increasing external Cd exposure and associated physiological disturbances, including oxidative stress and impairment of photosynthetic pigments [2,39]. These hydroponic treatments should not be interpreted as direct equivalents of specific soil Cd contents or field drought severity because PEG-induced dehydration does not reproduce all components of soil drought [37], whereas soil Cd bioavailability varies with physicochemical properties such as pH, organic matter content, and cation-exchange capacity and may exhibit substantial spatial heterogeneity [41]. Thus, the selected concentrations served primarily as physiologically discriminating screening levels rather than as exact simulations of field exposure.

Electrical conductivity, osmolality, and osmotic potential of the treatment solutions were not measured directly during the experiment. Therefore, the maximum theoretical osmotic contribution of CdCl_2_·2.5H_2_O was estimated using the van’t Hoff relation (Ψs = −iCRT). The elemental Cd concentrations of 5, 10, and 20 mg·L^−1^ corresponded to CdCl_2_·2.5H_2_O concentrations of 0.0445, 0.0890, and 0.1779 mmol·L^−1^, respectively. Assuming complete dissociation into one Cd^2+^ ion and two Cl^−^ ions (i = 3), the corresponding theoretical osmotic potentials were approximately −0.00033, −0.00066, and −0.00132 MPa at 25 °C. For comparison, the estimated osmotic potentials of the 5%, 7.5%, and 10% (*w*/*v*) PEG-6000 treatments were approximately −0.050, −0.093, and −0.148 MPa, respectively, based on the empirical equation of Michel and Kaufmann [40]. Thus, even at the highest Cd concentration, the maximum theoretical osmotic contribution of the added Cd salt was approximately 2.6% of that associated with the lowest PEG-6000 treatment and 0.9% of that associated with the highest PEG-6000 treatment. These estimates indicate that the additional osmotic contribution of the Cd salt was minor relative to that imposed by PEG-6000. However, this ideal calculation does not replace direct measurements and does not account for ion pairing or other non-ideal interactions in Hoagland solution; it addresses only the osmotic contribution of the added Cd salt rather than the intended ionic and toxic effects of Cd^2+^.

### 4.3. Measurement of Growth and Morphological Traits

After treatment, representative plants were selected from each replicate for morphological measurements. Plant height, maximum root length, minimum root length, maximum leaf length, minimum leaf length and stem length were measured using an electronic vernier caliper. Plant height was defined as the distance from the root-shoot junction to the highest point of the plant. Maximum and minimum root length referred to the longest root and the shorter young root within the same plant root system, respectively. Maximum and minimum leaf length referred to the elongation length of functional leaves and newly emerged young leaves, respectively. All length traits were expressed in cm.

### 4.4. Measurement of Biomass Allocation and Water Status

After morphological measurements, plants were separated into shoots and roots. Roots were quickly rinsed with deionized water and blotted dry with absorbent paper to remove surface water. Shoot fresh weight, root fresh weight and total fresh weight per plant were then measured using an analytical balance. Samples were dried at 65 °C to constant weight, and shoot dry weight and root dry weight were recorded. The root/shoot ratio was calculated as root dry weight divided by shoot dry weight. Shoot and root water content were calculated as follows: water content (%) = (fresh weight − dry weight)/fresh weight × 100%.

### 4.5. Determination of Photosynthetic Pigment Content

Chlorophyll and carotenoid contents were determined using 95% ethanol extraction [42]. For each treatment, 0.1 g of fresh seedling leaves was weighed, cut into small pieces, placed in a mortar, and ground rapidly under weak light with a small amount of quartz sand, calcium carbonate and 3 mL of 95% ethanol to minimize chlorophyll photooxidation. The homogenate was kept in the dark for 3–5 min and then centrifuged at 4000 r·min^−1^ for 5 min. The supernatant was transferred to a 25 mL brown volumetric flask. The precipitate was washed with a small amount of 95% ethanol, and the washing solution was combined with the supernatant and brought to volume with 95% ethanol. Absorbance was measured at 665, 649 and 470 nm.

Pigment concentrations were calculated using the following equations: chlorophyll a (Chl a) = 13.95A_665_ − 6.88A_649_; chlorophyll b (Chl b) = 24.96A_649_ − 7.32A_665_; total Chl = Chl a + Chl b; Car = (1000A_470_ − 2.05Chl a − 114.8Chl b)/245. Pigment content on a fresh-weight basis was calculated as: pigment content (mg·g^−1^ FW) = C × V × D/(1000 × W), where C is pigment concentration in the extract, V is the final volume (25 mL), D is the dilution factor (1 in this study) and W is leaf fresh weight (0.1 g). Chl a/b and Car/total Chl were also calculated to characterize photosynthetic pigment composition and photoprotective adjustment.

### 4.6. Determination of Malondialdehyde and Soluble Sugar Contents

Malondialdehyde (MDA) content and soluble sugar concentration were simultaneously determined using a two-component spectrophotometric method based on the thiobarbituric acid (TBA) reaction system [43]. Fresh leaf samples were ground in an ice bath with 100 g·L^−1^ trichloroacetic acid (TCA) and centrifuged. The supernatant was mixed with TBA reagent and incubated in a boiling water bath for color development. After the reaction, the mixture was rapidly cooled and centrifuged again. Absorbance was then measured at 450, 532 and 600 nm. Soluble sugar concentration was calculated as C_1_ = 11.71 × A450, where C_1_ represents soluble sugar concentration in mmol·L^−1^. MDA concentration was calculated as C_2_ = 6.45 × (A_532_ − A_600_) − 0.56 × A_450_, where C_2_ represents MDA concentration in μmol·L^−1^. Based on extract volume and sample fresh weight, MDA concentration was further converted to content per unit fresh weight and expressed as nmol·g−1 FW. A_450_, A_532_ and A_600_ represent absorbance of the reaction solution at 450, 532 and 600 nm, respectively.

### 4.7. Extraction of Antioxidant Enzymes and Determination of Enzyme Activities

Crude antioxidant enzyme extracts were prepared under low-temperature conditions, with slight modifications to published methods for POD and CAT activity determination [44,45]. Fresh leaves (0.1 g) were cut into pieces, placed in a pre-cooled mortar, and ground thoroughly in an ice bath with a small amount of quartz sand and 2 mL of pre-cooled phosphate extraction buffer (0.05 mol·L^−1^ KH_2_PO_4_-K_2_HPO_4_, pH 7.0, containing 0.1 mmol·L^−1^ ethylenediaminetetraacetic acid (EDTA) and 0.5 mmol·L^−1^ ascorbic acid). The homogenate was transferred to a centrifuge tube, brought to 5 mL with extraction buffer, and centrifuged at 12,000 r·min^−1^ for 15 min at 4 °C. The supernatant was used as the crude enzyme extract and kept on ice for POD and CAT assays.

Peroxidase (POD) activity was determined using guaiacol as the substrate. After preheating the reaction mixture, crude enzyme extract was added, and the change in absorbance at 470 nm was continuously recorded. One unit of POD activity was defined as an increase of 0.01 in A_470_ per minute. Catalase (CAT) activity was characterized by the degradation rate of H_2_O_2_. Briefly, 2.4 mL of 20 mmol·L^−1^ H_2_O_2_ solution was mixed with 0.4 mL of crude enzyme extract, and the decrease in absorbance at 240 nm was recorded. One unit of CAT activity was defined as a decrease of 0.01 in A_240_ per minute. Enzyme activities were calculated according to the absorbance change rate, total extraction volume, crude enzyme volume in the reaction system, and sample fresh weight and were expressed as U·g^−1^ FW·min^−1^.

### 4.8. Data Processing and Statistical Analysis

All data are presented as mean ± standard error (mean ± SE), with three biological replicates per treatment. The experiment used a three-factor full-factorial design with grass material (Grass), Cd concentration (0, 5, 10 and 20 mg·L^−1^) and PEG-6000 concentration (0%, 5%, 7.5%, and 10%, *w*/*v*) as fixed factors. Before statistical analysis, normality and homogeneity of variance were tested for each trait. To improve model residual distributions, raw data, log-transformed data, and square-root-transformed data were compared according to the characteristics of each trait, and the data form with better residual normality and variance homogeneity was preferentially selected for subsequent parametric analyses.

Three-way analysis of variance (three-way ANOVA) was used to test the effects of Grass, Cd, PEG and their interactions on each growth and physiological trait. Type III sums of squares were used in ANOVA, effect sizes were expressed as partial eta squared ($\eta_{p}^{2}$), and significance was set at *p* < 0.05. When the Grass × Cd × PEG interaction was significant, or when interpretation involved comparisons among grass materials within the same Cd × PEG treatment, simple-effect analysis was further conducted using estimated marginal means (EMMs). Specifically, within each Cd × PEG combination, pairwise comparisons among the three grasses were performed using grass material as the comparison factor. *p* values were adjusted by the Benjamini–Hochberg (BH) method, and compact letter displays were generated to indicate significance groups. This analysis was used to support judgments of significant differences among grass materials under the same stress condition.

To further show trait responses among different Cd and PEG-6000 treatment combinations within each grass, Cd concentration and PEG-6000 concentration were combined into 16 Cd × PEG treatment levels, and treatment comparisons were conducted separately within each grass material. Because some traits still did not simultaneously meet the assumptions of normality and homogeneity of variance required for parametric post hoc comparisons after transformation, and because this analysis was mainly intended to show differences among treatment combinations within the same grass, the Kruskal–Wallis test was used as a complementary non-parametric analysis for within-species treatment differences. When significant, Dunn’s post hoc test was used for pairwise comparisons, with *p* values adjusted by the BH method. This non-parametric comparison was used only to assess differences among Cd × PEG combinations for the same trait within the same grass, not to replace the three-way Grass × Cd × PEG ANOVA or to directly determine significant differences among grasses within the same Cd × PEG treatment.

To determine whether combined Cd and PEG-6000 stress tended to enhance membrane lipid peroxidation, MDA content was used as a representative trait to calculate the theoretical additive value of the combined stress and the synergistic damage coefficient. For the same grass material and the same Cd × PEG combination, the theoretical additive MDA value was estimated by adding the single-Cd effect and the single-PEG-6000 effect as follows:

$${MDA}_{add}=\;{MDA}_{Cd}+\;{MDA}_{PEG}-\;{MDA}_{CK}$$

where ${MDA}_{add}$ is the theoretical value obtained by adding the effects of the two single stresses; ${MDA}_{Cd}$ is the MDA content under the corresponding Cd concentration with 0% (*w*/*v*) PEG-6000; ${MDA}_{PEG}$ is the MDA content under 0 mg·L^−1^ Cd with the corresponding PEG-6000 concentration; and ${MDA}_{CK}$ is the MDA content under the control treatment (0 mg·L^−1^ Cd + 0% (*w*/*v*) PEG-6000). The synergistic damage coefficient was then calculated as:

$$K\;=\;{MDA}_{obs}/{MDA}_{add}$$

where ${MDA}_{obs}$/ is the measured MDA value under the Cd × PEG combined treatment. When K > 1, the measured MDA under combined stress is higher than the theoretical additive value, indicating enhanced membrane lipid peroxidation or a synergistic damage tendency. When K ≈ 1, the response is considered approximately additive; when K < 1, an antagonistic effect may be present. This calculation was used only to assist interpretation of combined-stress damage at the MDA level and did not replace the statistical tests of Cd, PEG-6000 and their interaction in the three-way ANOVA.

To avoid interpretive bias caused by changes in tissue water status and reduced shoot fresh weight when photosynthetic pigments are expressed on a fresh-weight basis, the relative amount of shoot total chlorophyll under severe combined stress was further estimated. Because chlorophyll content was measured per unit fresh weight, shoot total chlorophyll amount was calculated for each biological replicate as the product of total chlorophyll concentration per unit fresh weight and shoot fresh weight: shoot total Chl = total Chl × shoot FW/1000, where shoot total Chl is the estimated amount of total chlorophyll in the shoot (mg), total Chl is total chlorophyll concentration on a fresh-weight basis (mg g^−1^ FW), shoot FW is shoot fresh weight (mg), and division by 1000 converts mg fresh weight to g fresh weight. The relative shoot total chlorophyll amount was then calculated using the species-specific control as the baseline: relative shoot total Chl amount (%) = shoot total Chlstress/shoot total Chlcontrol × 100%. This derived trait was calculated from raw replicate data and used only to assist interpretation of changes in chlorophyll on a fresh-weight basis; it was not treated as an independent measured trait for significance testing.

To improve the robustness of integrated tolerance evaluation, stress tolerance coefficient (STC), membership function analysis and principal component analysis (PCA) were combined for cross-validation [46]. STC was calculated relative to the species-specific control treatment (0 mg·L^−1^ Cd + 0% (*w*/*v*) PEG-6000). For positive traits, for which larger values generally indicate better growth maintenance or physiological stability, STC was calculated as the ratio of the stress-treatment mean to the corresponding species-specific control mean. For the negative trait MDA, STC was calculated as the ratio of the corresponding species-specific control mean to the stress-treatment mean. In this way, all STC values were oriented such that larger values indicated stronger relative tolerance. Plant height, maximum root length, minimum root length, maximum leaf length, minimum leaf length, stem length, shoot fresh weight, root fresh weight, shoot dry weight, root dry weight, shoot water content, root water content, Chl a, Chl b, carotenoids, total chlorophyll, POD, CAT and soluble sugars were treated as positive traits, whereas MDA was treated as a negative trait.

Membership function analysis was then performed based on STC values. For each Cd × PEG combination and each trait, STC values were normalized to a 0–1 scale among the three grasses using the following equation:

$$U\;=\;\frac{STC\;-\;{STC}_{min}}{{STC}_{max}\;-\;{STC}_{min}}$$

where U is the membership function value, and X_min_ and X_max_ are the minimum and maximum STC values among the three grasses under the same Cd × PEG treatment for the same trait. When X_max_ = X_min_, U was assigned a value of 0.5. The mean U value across all traits was then calculated for each species under each Cd × PEG combination and used as the integrated membership function score.

PCA was conducted using 48 treatment means, corresponding to 3 grass materials × 4 Cd concentrations × 4 PEG-6000 concentrations. Before analysis, all traits were standardized by z-score transformation to remove scale effects. For the negative damage trait MDA, direction correction was applied before PCA so that its direction was consistent with tolerance. PC1 and PC2 were used mainly to visualize the distribution of different treatments in multivariate trait space and were not used alone for tolerance ranking. For integrated PCA evaluation, PCA scores were further calculated using the first five principal components and their variance contributions, and component directions were corrected so that larger values indicated more favorable integrated multi-trait performance.

Finally, the STC mean, membership function mean and PCA integrated score were standardized and averaged to obtain a multi-method integrated tolerance score. Within each Cd × PEG treatment combination, the three grasses were then ranked according to the integrated score. Interpretation of comprehensive tolerance did not rely on a single statistical index but jointly considered STC, membership function values, PCA-integrated scores, and individual growth and physiological traits.

Pearson correlation analysis was used to assess relationships among traits, and a correlation heatmap was generated to show synergistic or antagonistic associations. Significance levels were defined as follows: * *p* < 0.05, ** *p* < 0.01, *** *p* < 0.001 and ns, not significant (*p* ≥ 0.05). All statistical analyses and plots were completed in the R environment using the tidyverse, car, emmeans, multcomp, effectsize, rstatix, ARTool, openxlsx, ggplot2 and factoextra packages.

## 5. Conclusions

This study demonstrated species-specific changes in growth, biomass allocation, water status, membrane lipid peroxidation, osmotic adjustment, antioxidant enzyme activities, and photosynthetic pigments under factorial combinations of nominal external Cd concentrations and PEG-6000-induced osmotic stress. Under the most severe treatment, *E. breviaristatus* retained more plant height and shoot dry matter, had a lower absolute MDA content, and maintained relatively high CAT activity and chlorophyll concentration. It also ranked first in the phenotype-based multi-trait evaluation, indicating comparatively favorable performance under the tested hydroponic seedling conditions. However, because Cd availability, root and shoot Cd concentrations and total contents, and root-to-shoot translocation were not quantified, this ranking cannot distinguish Cd exclusion, root retention, reduced translocation, or intrinsic tissue tolerance. Therefore, *E. breviaristatus* should be regarded as a candidate for further validation rather than as a confirmed Cd-tolerant or field-restoration material. Future studies should quantify Cd uptake and distribution and verify these responses in soil-based and multi-year field experiments.

## Figures and Tables

**Table 1:** Integrated ranking of the measured multi-trait responses of three *Elymus* grasses under severe combined treatment.

Species	STC Mean	STC Rank	Membership Mean	Membership Rank	PCA Score	PCA Rank	Integrated Score	Integrated Rank
*E. breviaristatus*	1.168	1	0.612	1	0.603	1	0.692	1
*E. nutans*	1.005	2	0.523	2	−0.723	2	−0.219	2
*E. sibiricus*	0.860	3	0.423	3	−1.888	3	−1.089	3

Note: STC, stress tolerance coefficient. Membership mean represents the average membership function value across traits. PCA score was calculated from the first five principal components after direction correction. Integrated score was calculated from standardized STC mean, membership mean and PCA score. All values were calculated under 20 mg·L^−1^ Cd + 10% (*w*/*v*) PEG-6000 severe co-stress. Higher values indicate more favorable relative multi-trait performance under the tested external treatment conditions. This phenotype-based ranking does not account for differences in Cd uptake, tissue Cd concentrations, root retention, or root-to-shoot translocation and should not be interpreted as a direct ranking of intrinsic tissue Cd tolerance.

## Data Availability

The original contributions presented in this study are included in the article/Supplementary Material. Further inquiries can be directed to the corresponding author.

## Supplementary Materials

The following Supporting Information can be downloaded at: https://www.mdpi.com/article/10.3390/plants15162434/s1, Figure S1: Detailed grouped bar plots of growth and morphological responses of three *Elymus* grasses under the Cd × PEG-6000 treatments. Values are means ± SE (n = 3). Different lowercase letters indicate significant differences among treatment combinations within each grass species at *p* < 0.05. Figure S2: Detailed grouped bar plots of biomass allocation responses of three Elymus grasses under the Cd × PEG-6000 treatments. Values are means ± SE (n = 3). Different lowercase letters indicate significant differences among treatment combinations within each grass species at *p* < 0.05. FW, fresh weight; DW, dry weight. Figure S3: Chlorophyll a, chlorophyll b, and carotenoid responses of three *Elymus* grasses under combined cadmium and PEG-6000-induced osmotic stress. Values are means ± SE (n = 3). Chl a, chlorophyll a; Chl b, chlorophyll b. Table S1: Complete three-way ANOVA results for the effects of grass species, Cd concentration, PEG-6000 concentration, and their interactions on all measured traits.

## References

[1] Seleiman M.F., Al-Suhaibani N., Ali N., Akmal M., Alotaibi M., Refay Y., Dindaroglu T., Abdul-Wajid H.H., Battaglia M.L. (2021). Drought Stress Impacts on Plants and Different Approaches to Alleviate Its Adverse Effects. Plants.

[2] Luo P., Wu X., Li T.T., Shi P.H., Ma Q., Di D.W. (2024). An Overview of the Mechanisms through Which Plants Regulate ROS Homeostasis under Cadmium Stress. Antioxidants.

[3] Yang Q., Wang S., Zhao C., Nan Z. (2022). Risk Assessment of Trace Elements Accumulation in Soil–Herbage Systems at Varied Elevation in Subalpine Grassland of Northern Tibet Plateau. Environ. Sci. Pollut. Res..

[4] Nadeem H., Khan A., Gupta R., Hashem M., Alamri S., Siddiqui M.A., Ahmad F. (2023). Stress Combination: When Two Negatives May Become Antagonistic, Synergistic or Additive for Plants?. Pedosphere.

[5] Qi Y., Ma L., Ghani M.I., Peng Q., Fan R., Hu X., Chen X. (2023). Effects of Drought Stress Induced by Hypertonic Polyethylene Glycol (PEG-6000) on *Passiflora edulis* Sims Physiological Properties. Plants.

[6] Ma Y., Hu J.-C., Yu Y., Cheng X., Du Y.-L., Zhao Q., Du J.-D. (2024). Interactive Effects of Drought and Cadmium Stress on Adzuki Bean Seedling Growth, DNA Damage Repair, and Cd Accumulation. Sci. Hortic..

[7] Wang X., Hu Z., Zhang Z., Tang J., Niu B. (2024). Altitude-Shifted Climate Variables Dominate the Drought Effects on Alpine Grasslands over the Qinghai–Tibetan Plateau. Sustainability.

[8] Li J., Ma S., Jiang K., Zhang C., Liu W., Chen S. (2023). Inflorescence Trait Diversity and Genotypic Differentiation as Influenced by the Environment in *Elymus nutans* Griseb. from Qinghai–Tibet Plateau. Agronomy.

[9] Wu R., Liu W.H., Zhang Y.C., Liang G.L., Li W., Liu K.Q. (2024). Response of *Elymus sibiricus* (Siberian Wildryegrass) to Combined Application of Nitrogen and Phosphorus during Aging on the Qinghai–Tibetan Plateau. Agronomy.

[10] Yu Q., Liu Q., Xiong Y., Xiong Y., Dong Z., Yang J., Liu W., Ma X., Bai S. (2019). Genetic Diversity and Population Divergence of a Rare, Endemic Grass (*Elymus breviaristatus*) in the Southeastern Qinghai-Tibetan Plateau. Sustainability.

[11] Suzuki N., Rivero R.M., Shulaev V., Blumwald E., Mittler R. (2014). Abiotic and biotic stress combinations. New Phytol..

[12] Gallego S.M., Pena L.B., Barcia R.A., Azpilicueta C.E., Iannone M.F., Rosales E.P., Zawoznik M.S., Groppa M.D., Benavides M.P. (2012). Unravelling cadmium toxicity and tolerance in plants: Insight into regulatory mechanisms. Environ. Exp. Bot..

[13] Zhang H., Sonnewald U. (2017). Differences and commonalities of plant responses to single and combined stresses. Plant J..

[14] Anwar T., Shehzadi A., Qureshi H., Shah M.N., Danish S., Salmen S.H., Ansari M.J. (2023). Alleviation of cadmium and drought stress in wheat by improving growth and chlorophyll contents amended with GA3 enriched deashed biochar. Sci. Rep..

[15] Li M.Q., Yang J., Wang X., Li D.X., Zhang C.B., Tian Z.H., You M.H., Bai S.Q., Lin H.H. (2020). Transcriptome profiles identify the common responsive genes to drought stress in two *Elymus* species. J. Plant Physiol..

[16] Zhang X.X., Li C.J., Nan Z.B. (2012). Effects of cadmium stress on seed germination and seedling growth of *Elymus dahuricus* infected with the Neotyphodium endophyte. Sci. China Life Sci..

[17] Guasconi D., Manzoni S., Hugelius G. (2023). Climate-dependent responses of root and shoot biomass to drought duration and intensity in grasslands—A meta-analysis. Sci. Total Environ..

[18] Freschet G.T., Roumet C., Comas L.H., Weemstra M., Bengough A.G., Rewald B., Bardgett R.D., De Deyn G.B., Johnson D., Klimešová J. (2021). Root traits as drivers of plant and ecosystem functioning: Current understanding, pitfalls and future research needs. New Phytol..

[19] Poschenrieder C., Gunsé B., Barceló J. (1989). Influence of Cadmium on Water Relations, Stomatal Resistance, and Abscisic Acid Content in Expanding Bean Leaves. Plant Physiol..

[20] Perfus-Barbeoch L., Leonhardt N., Vavasseur A., Forestier C. (2002). Heavy Metal Toxicity: Cadmium Permeates through Calcium Channels and Disturbs the Plant Water Status. Plant J..

[21] Belimov A.A., Dodd I.C., Safronova V.I., Malkov N.V., Davies W.J., Tikhonovich I.A. (2015). The Cadmium-Tolerant Pea (*Pisum sativum* L.) Mutant SGECdᵗ Is More Sensitive to Mercury: Assessing Plant Water Relations. J. Exp. Bot..

[22] Andrade Júnior W.V., de Oliveira Neto C.F., dos Santos Filho B.G., do Amarante C.B., Cruz E.D., Okumura R.S., Barbosa A.V.C., de Sousa D.J.P., Teixeira J.S.S., de Santana Botelho A. (2019). Effect of Cadmium on Young Plants of *Virola surinamensis*. AoB Plants.

[23] Liu Z., Hou L., Yan J., Ahmad P., Qin M., Li R., El-Sheikh M.A., Deshmukh R., Sudhakaran S.S., Ali B. (2024). Aquaporin mediated silicon-enhanced root hydraulic conductance is benefit to cadmium dilution in tobacco seedlings. J. Hazard. Mater..

[24] Gill S.S., Tuteja N. (2010). Reactive oxygen species and antioxidant machinery in abiotic stress tolerance in crop plants. Plant Physiol. Biochem..

[25] Morales M., Munné-Bosch S. (2019). Malondialdehyde: Facts and artifacts. Plant Physiol..

[26] Sami F., Yusuf M., Faizan M., Faraz A., Hayat S. (2016). Role of sugars under abiotic stress. Plant Physiol. Biochem..

[27] Tao J., Lu L. (2022). Advances in Genes-Encoding Transporters for Cadmium Uptake, Translocation, and Accumulation in Plants. Toxics.

[28] Chaves M.M., Flexas J., Pinheiro C. (2009). Photosynthesis under drought and salt stress: Regulation mechanisms from whole plant to cell. Ann. Bot..

[29] Haider F.U., Liqun C., Coulter J.A., Cheema S.A., Wu J., Zhang R., Wenjun M., Farooq M. (2021). Cadmium toxicity in plants: Impacts and remediation strategies. Ecotoxicol. Environ. Saf..

[30] Zhao H., Guan J., Liang Q., Zhang X., Hu H., Zhang J. (2021). Effects of cadmium stress on growth and physiological characteristics of sassafras seedlings. Sci. Rep..

[31] Yan W.J., Lu Y.C., Guo L.C., Liu Y., Li M.K., Zhang B.Y., Zhang B.X., Zhang L.J., Qin D., Huo J.W. (2024). Effects of Drought Stress on Photosynthesis and Chlorophyll Fluorescence in Blue Honeysuckle. Plants.

[32] Liu J., Ni J., Mo A., Fan X.T., Jiang Y.Y., Xie H.Y., Hu J.S., Zhu Y.H., Peng C.Y., Yang E. (2023). Cadmium Affects the Growth, Antioxidant Capacity, Chlorophyll Content, and Homeostasis of Essential Elements in Soybean Plants. S. Afr. J. Bot..

[33] Mishra N., Jiang C., Chen L., Paul A., Chatterjee A., Shen G. (2023). Achieving Abiotic Stress Tolerance in Plants through Antioxidative Defense Mechanisms. Front. Plant Sci..

[34] Dong Q., Xu P., Wang Z. (2017). Differential Cadmium Distribution and Translocation in Roots and Shoots Related to Hyper-Tolerance between Tall Fescue and Kentucky Bluegrass. Front. Plant Sci..

[35] Wang J., Zhao J., Feng S., Zhang J., Gong S., Qiao K., Zhou A. (2020). Comparison of Cadmium Uptake and Transcriptional Responses in Roots Reveals Key Transcripts from High- and Low-Cadmium-Tolerance Ryegrass Cultivars. Ecotoxicol. Environ. Saf..

[36] Miyadate H., Adachi S., Hiraizumi A., Tezuka K., Nakazawa N., Kawamoto T., Katou K., Kodama I., Sakurai K., Takahashi H. (2011). OsHMA3, a P1B-Type ATPase, Affects Root-to-Shoot Cadmium Translocation in Rice by Mediating Efflux into Vacuoles. New Phytol..

[37] Kylyshbayeva G., Bishimbayeva N., Jatayev S., Eliby S., Shavrukov Y. (2025). Polyethylene Glycol (PEG) Application Triggers Plant Dehydration but Does Not Accurately Simulate Drought. Plants.

[38] Verslues P.E., Agarwal M., Katiyar-Agarwal S., Zhu J., Zhu J.K. (2006). Methods and concepts in quantifying resistance to drought, salt and freezing, abiotic stresses that affect plant water status. Plant J..

[39] Hu Y., Wang H., Jia H., Peng M., Zhu T., Liu Y., Wei J. (2023). Effects of Cd treatment on morphology, chlorophyll content and antioxidant enzyme activity of *Elymus nutans* Griseb., a native plant in Qinghai-Tibet Plateau. Plant Signal. Behav..

[40] Michel B.E., Kaufmann M.R. (1973). The Osmotic Potential of Polyethylene Glycol 6000. Plant Physiol..

[41] Wang F., Huang C., Chen Z., Bao K. (2019). Distribution, Ecological Risk Assessment, and Bioavailability of Cadmium in Soil from Nansha, Pearl River Delta, China. Int. J. Environ. Res. Public Health.

[42] Li J., Lian X., Ye C., Wang L. (2019). Analysis of flower color variations at different developmental stages in two honeysuckle (*Lonicera japonica* Thunb.) cultivars. HortScience.

[43] Cai M.L., Zhang Q.L., Zheng X.T., Zhai J.J., Peng C.L. (2020). Comparison of leaves and stems of *Paederia scandens* (Lour.) Merr. in tolerance to low temperature. Photosynthetica.

[44] Chance B., Maehly A.C. (1955). Assay of catalases and peroxidases. Methods Enzymol..

[45] Aebi H. (1984). Catalase in vitro. Methods Enzymol..

[46] Yan C., Song S., Wang W., Wang C., Li H., Wang F., Li S., Sun X. (2020). Screening diverse soybean genotypes for drought tolerance by membership function value based on multiple traits and drought-tolerant coefficient of yield. BMC Plant Biol..

