# Supplementary material for: Contrasting Growth and Physiological Response Profiles of Three Native Alpine Elymus Forage Grasses Under Combined Cadmium and PEG-6000-Induced Osmotic Stress
Source: Plants (Basel). 2026 Aug 10;15(16):2434. doi: 10.3390/plants15162434 (PMC13516643; doi:10.3390/plants15162434)
Supplement: Supplementary file 1 [file plants-15-02434-s001.zip › Supplementary Figures.pdf]

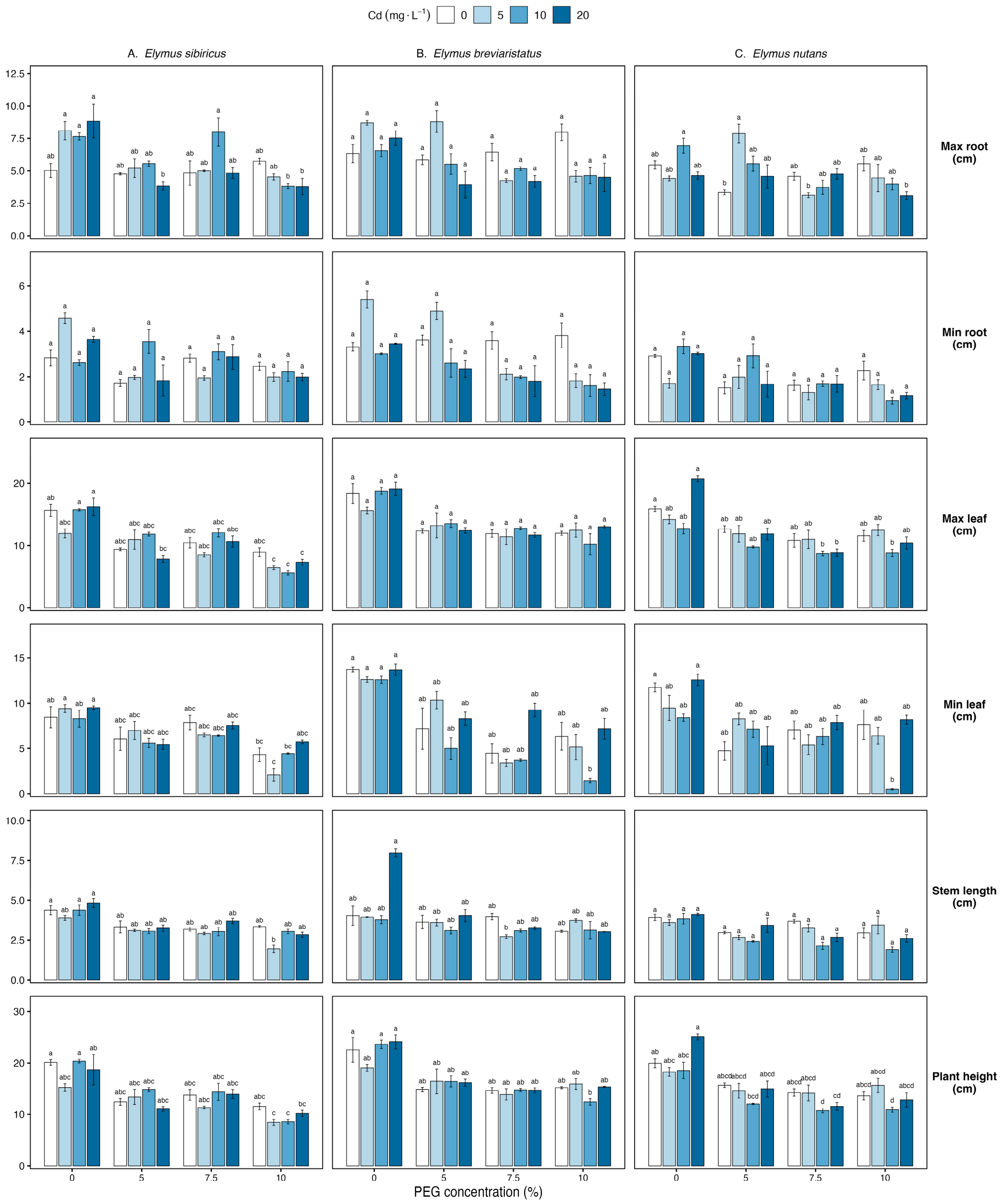

Figure S1. Detailed grouped-bar plots of growth and morphological responses of three *Elymus* grasses under the Cd × PEG-6000 treatments. Values are means ± SE (n = 3). Different lowercase letters indicate significant differences among treatment combinations within each grass species at $P < 0.05$.

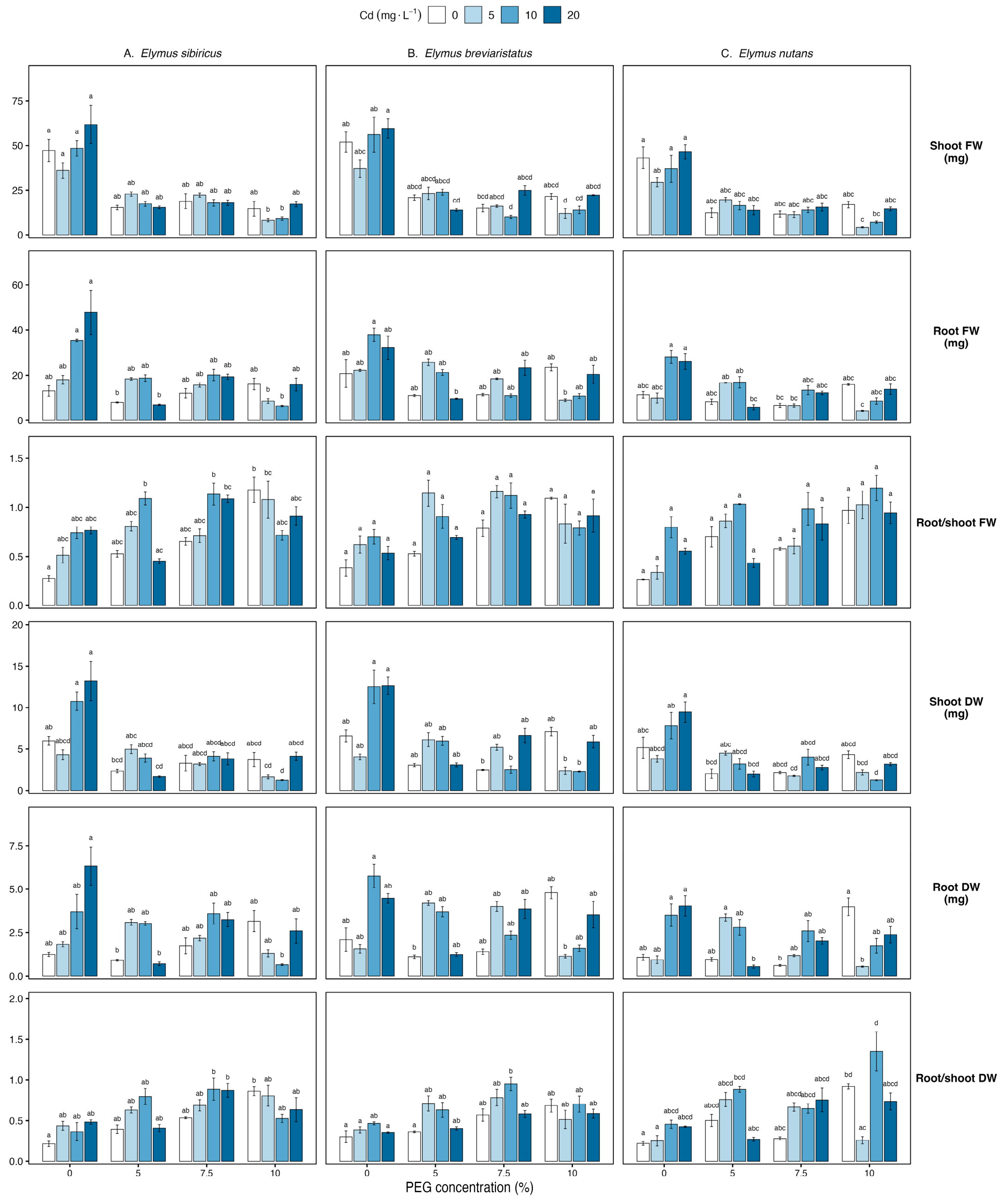

Figure S2. Detailed grouped-bar plots of biomass allocation responses of three Elymus grasses under the Cd × PEG-6000 treatments. Values are means ± SE (n = 3). Different lowercase letters indicate significant differences among treatment combinations within each grass species at P < 0.05. FW, fresh weight; DW, dry weight.

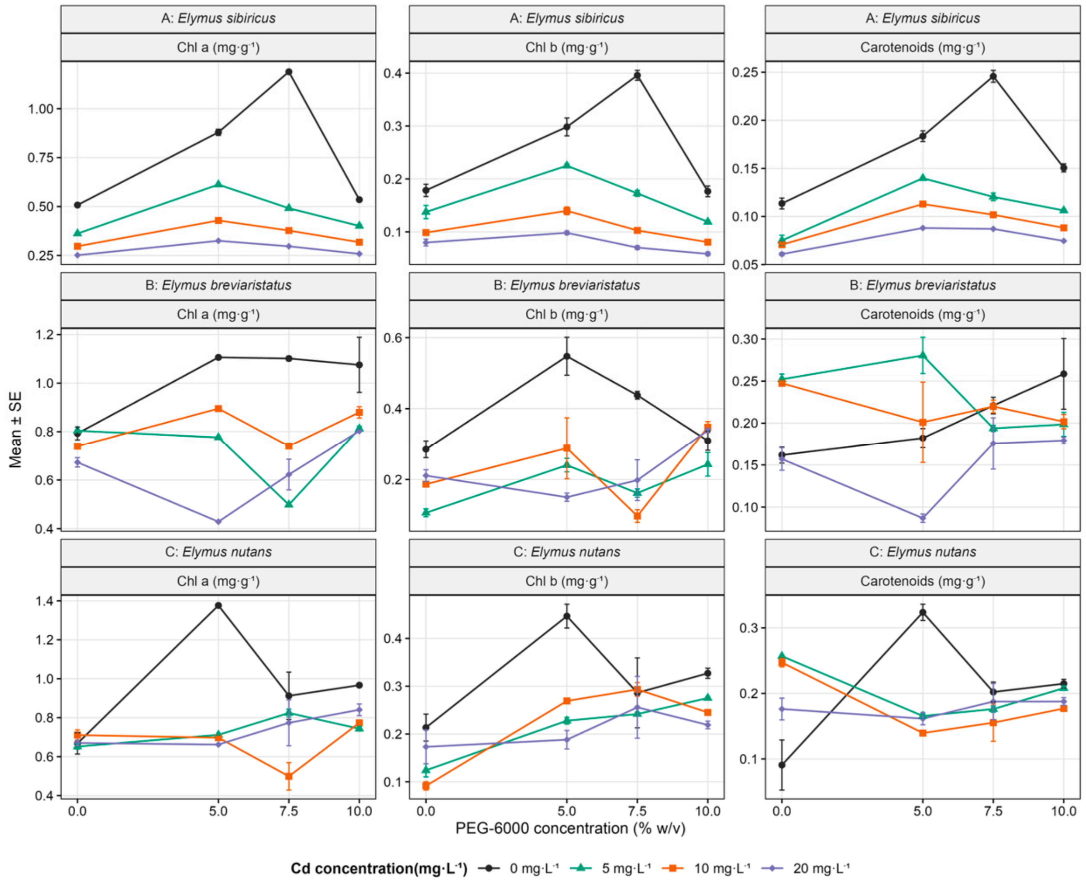

Figure S3. Chlorophyll a, chlorophyll b, and carotenoid responses of three *Elymus* grasses under combined cadmium and PEG-6000-induced osmotic stress. Values are means ± SE (n = 3). Chl a, chlorophyll a; Chl b, chlorophyll b.
